# Feature-Level Decoupling of Reinforced-Concrete Parameters from Sparse MFL Data via Three-Layer Sensing

**DOI:** 10.3390/s26217049

**Published:** 2026-11-07

**Authors:** Paweł Karol Frankowski, Cindy Almosura Lasco, Alan Karl Syting Gatdula, Marcin Mąka, Ryszard Daniel Ziętek, Radosław Gordon, Paweł Owczarek, Patryk Pocheć, Tomasz Stawicki, Sebastian Matysik

**Affiliations:** 1 Department of Information and Communication Technologies, Faculty of Computer Science and Telecommunications, Maritime University of Szczecin, Wały Chrobrego 1-2, 70-500 Szczecin, Poland; m.maka@pm.szczecin.pl; 2 Faculty of Computing, Engineering and Technology, Davao Oriental State University, Guang-guang, Dahican, City of Mati 8200, Davao Oriental, Mindanao, Philippines; cindy.lasco@dorsu.edu.ph (C.A.L.); alankarl.gatdula@dorsu.edu.ph (A.K.S.G.); 3 Department of Robotics and Control, Faculty of Mechatronics and Electrical Engineering, Maritime University of Szczecin, Willowa 2, 71-650 Szczecin, Poland; r.zietek@pm.szczecin.pl; 4 Department of Electrical Engineering and Power Electronics, Faculty of Mechatronics and Electrical Engineering, Maritime University of Szczecin, Willowa 2, 71-650 Szczecin, Poland; r.gordon@pm.szczecin.pl; 5 Student Science Club KN BIT, Faculty of Computer Science and Telecommunications, Maritime University of Szczecin, Wały Chrobrego 1-2, 70-500 Szczecin, Poland; 31350@s.pm.szczecin.pl (P.O.); 31339@s.pm.szczecin.pl (P.P.); 6 Department of Engineering of Renewable Energy Sources, Faculty of Environmental Management and Agriculture, West Pomeranian University of Technology in Szczecin, Papieza Pawla VI Street 1, 71-459 Szczecin, Poland; tomasz.stawicki@zut.edu.pl; 7 Doctoral School, University of Szczecin, Mickiewicza 18, 70-384 Szczecin, Poland; sebastian.matysik@usz.edu.pl; 8 Institute of Management, University of Szczecin, Cukrowa 8, 71-004 Szczecin, Poland

**Keywords:** parameter decoupling, magnetic flux leakage, non-destructive testing, reinforced concrete, association rule analysis, explainable artificial intelligence, few-shot data learning, sparse data, signal processing, technology acceptance

## Abstract

**Highlights:**

- Three-layer MFL sensing decouples rebar diameter, cover thickness, and steel class.
- ACO, association rules, and rough sets yield human-readable feature-parameter rules.
- Waveform features, each sensitive to only one RC parameter, support sparse-data NDT.
- Rough-set bounds expose uncertainty and support auditable glass-box decisions.
- The method needs only a few reference measurements, not large labeled datasets.

**Abstract:**

This methodological paper presents a data-efficient, explainable artificial intelligence (XAI) pipeline for non-destructive testing (NDT) of reinforced concrete (RC) under sparse-data conditions, addressing three closely connected challenges: cross-parameter interference in magnetic flux leakage (MFL) signals, scarce training data, and black-box opacity. Data scarcity is a recognized constraint across AI-driven NDT and is structural in reinforced concrete (costly labeling, absent benchmarks, small-data learning missing from the reviewed electromagnetic literature). This scarcity has two consequences: black-box models can generalize poorly on sparse data, and where errors are likely, engineers hesitate to trust or assume liability for opaque predictions. We present a glass-box framework identifying rebar diameter (*D*), cover thickness (*h*), and steel class through feature-level decoupling grounded in measurement physics. Transparency and accuracy are treated as equally vital NDT dimensions. Amplitude–Correlation–Offset (ACO) decomposition of a three-layer magnetoresistive transducer isolates parameter-specific attributes: *B*_x_ offset classifies steel class (95% confidence), genetic-algorithm fitting localizes peaks to 0.1 mm for *D* (10–12 mm), and inter-layer amplitude ratios estimate *h* (20–60 mm). Rough-set analysis quantifies rule confidence and uncertainty zones. Proof-of-concept validation (20 specimens and 400 measurements) shows that a few reference configurations suffice for interpretable identification, enabling explainable artificial intelligence (XAI) in sparse-data NDT and lowering the technology-adoption barrier in safety-critical inspection.

## 1. Introduction

This section explains the motivation and background for the work, including a systematic literature review of artificial intelligence (AI) methods used in non-destructive testing (NDT) of civil infrastructure. The review data were collected from Scopus on 13 August 2026 via a structured query (Supplementary Section S1). It yielded 235 records, which were screened using an embedding-based method [1] to identify the most relevant studies for the research questions. While no single Scopus search can capture all relevant publications, this corpus offers a structured, reproducible snapshot of the available literature.

### 1.1. Motivation

Magnetic flux leakage (MFL) inspection of reinforced-concrete (RC) structures produces signals influenced by multiple parameters, such as reinforcing-bar diameter, steel class, and concrete cover thickness, complicating cause attribution [2]. Traditional analysis methods that examine multiple features tend to overfit, require extensive data across parameters (rare in practice), and struggle with nonlinear relationships and variables such as permeability, bar positioning, and inhomogeneity [3,4,5]. Small datasets hinder generalization, increase sensitivity to noise, and make importance rankings less stable, thereby reducing interpretability [6,7].

Accuracy alone cannot ensure AI adoption in safety-critical inspections. Opaque models undermine trust and complicate interpretation, making engineers and stakeholders hesitant to rely on AI [8,9,10], particularly in critical safety assessments [11]. Despite AI’s advances, RC inspections often still require costly, invasive concrete removal when specifications or documentation are missing. AI has the potential to speed up testing and lower costs, while maintaining safety. Explainable AI (XAI) addresses this by making models more understandable [10,12]; in NDT, it clarifies decisions, strengthening validation and confidence [13]. This transparency builds trust and helps overcome psychological barriers. In regulated inspections, adoption is as much a technology-acceptance issue as a technical one: key criteria include perceived usefulness and verifiability by the responsible engineer. Transparency is therefore a design requirement, not merely a desirable by-product [10,13].

Interest in small-data and few-shot learning grew steadily from 2017 and exploded in 2025, when search volume doubled, relative to 2024 (Figure S11). Partial 2026 data point to a further acceleration: if the trend holds, search volume will increase not twofold, but fourfold, over 2025. These worldwide trends reflect interest not only from researchers, but also from engineers and developers working with limited datasets in practice. However, this interest is not reflected in the reviewed NDT corpus: records addressing data scarcity remain a small share of the output, rising from 1.7% in 2005–2019 to 7.4% in 2020–2026 (Figure S14).

The reviewed corpus (Section 2.2), nevertheless, shows rapid growth in the method families this work builds on. Multitask learning, motivated by the practical need to identify several parameters at once, grew from 2.1% of records in 2020–2022 to 24.7% in 2025–2026, with explicit decoupling at 20.8% and rule-based approaches at 15.6%; all three were near-zero before 2015 (Table S2). This multi-parameter setting, in turn, increases the data demand of most classical methods (the curse of dimensionality), compounding the sparse-data challenge, and making it the structural constraint behind this work.

Meanwhile, publication output in the two broader fields has plateaued: NDT for concrete grew approximately fourfold between 2001 and 2018, and has since leveled off (Figure S12), and machine-learning research for predictive modeling shows a similar pattern, with sustained output above 3000 publications per year since 2010 (Figure S13). The broader fields are therefore mature, while the specific methods this work builds on are still expanding rapidly. Their intersection, however, remains largely unexplored: no study in the reviewed corpus of 235 publications combines small-data learning with an electromagnetic modality (Section 2.2). This work addresses that gap as a methodological case study, demonstrating a data-efficient, interpretable pipeline for identifying reinforced-concrete parameters.

Parameter coupling is the specific obstacle this work targets. The proposed method decouples parameters via Association Rule Analysis (ARA) [14], identifying signal features that respond primarily to one physical parameter and remain invariant to others, reducing the three-parameter problem to three single-parameter tasks. Unlike latent-space disentanglement in deep learning [15], decoupling occurs directly at the feature level using human-readable rules, ensuring transparency and less post hoc explanation [16]. Feature-level decoupling associates each parameter with an interpretable signal feature that is primarily sensitive to it and relatively invariant to others, though identification is not perfect. The term denotes the method’s design principle; the invariance demonstrated in this study holds within the investigated experimental conditions, and its extension beyond them is a physical expectation, rather than an experimental result. The method combines ARA [14], Amplitude–Correlation–Offset (ACO) decomposition [17], rough sets, and genetic-algorithm curve fitting [18], with a modular design that enhances noise robustness and confines errors to individual parameters [19].

Parameter decoupling is vital in NDT, where testing all parameter combinations is impractical. The rule-based approach links structural changes to signal variations and physical phenomena, providing feedback for modeling and simulating complex objects.

The proposed method is designed to use human-readable rules to connect signal changes to structural parameters, and embodies the “glass-box” principle. As infrastructure ages, smart, AI-assisted NDT research is crucial, transforming construction, supporting smart-city initiatives, and enabling robust, interpretable parameter identification from field data.

### 1.2. Artificial Intelligence and Machine Learning in Non-Destructive Testing

This section reviews AI and machine learning (ML) in NDT, focusing on data-driven inspection of aging RC infrastructure, method families, applications to RC parameter identification, open challenges, and parameter decoupling.

#### 1.2.1. Introduction to AI/ML in NDT

**Figure 1:**
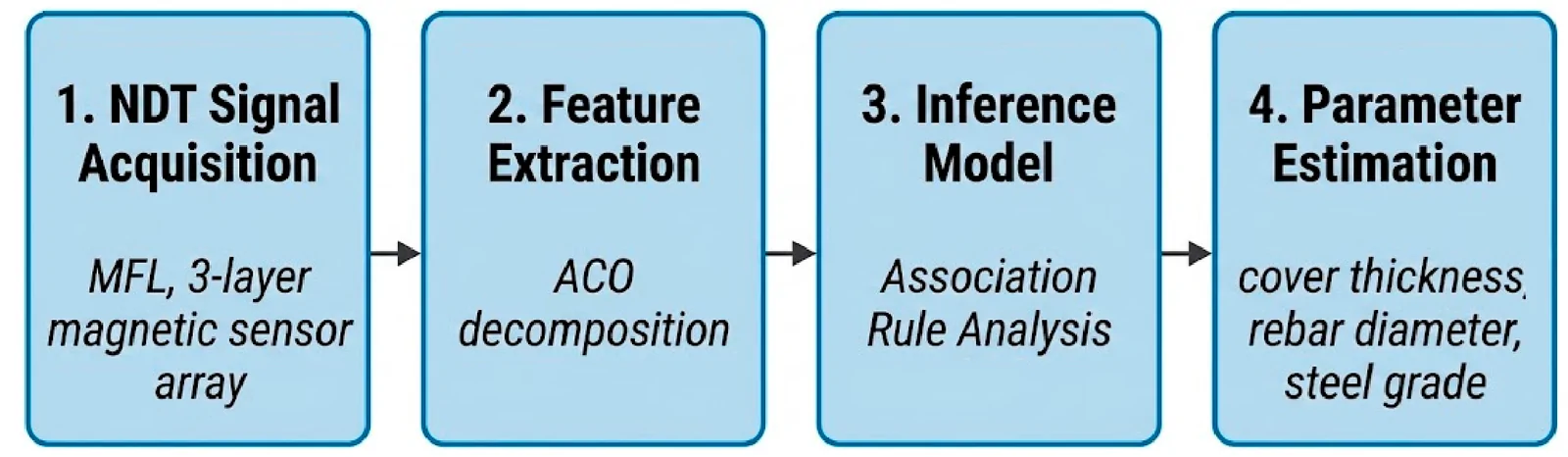
Four-stage AI-NDT pipeline with example implementations used in this study.

Civil infrastructure is aging, and inspection needs for RC bridges, buildings, and tunnels are growing faster than the pool of qualified inspectors [20,21]. Conventional RC tests are slow, often destructive, and operator-dependent; field campaigns show that protocol variability and sensor performance limit rapid assessment [3,4]. AI and ML enable automation and pattern detection beyond human perception [21,22], with data-driven pipelines covering the entire concrete structure lifecycle [19,23] and extensive adoption in RC [5]. Two key caveats remain: high-performing models require large datasets, but labeled RC data are scarce [24]—a structural constraint, since preparing a single specimen takes weeks (Section 3.2.1). These models also act as black boxes, raising safety concerns [16,25]. A typical AI-NDT pipeline has four stages (Figure 1); this study addresses all four, from MFL signal acquisition through feature extraction and inference to parameter estimation.

#### 1.2.2. Overview of AI/ML Methods in NDT

Method families are organized based on the type of data they process, as this choice (rather than the learning algorithm) determines which physical quantities can be recovered. Table 1 categorizes these families and highlights their main advantages and limitations in NDT applications.

**Figure 2:**
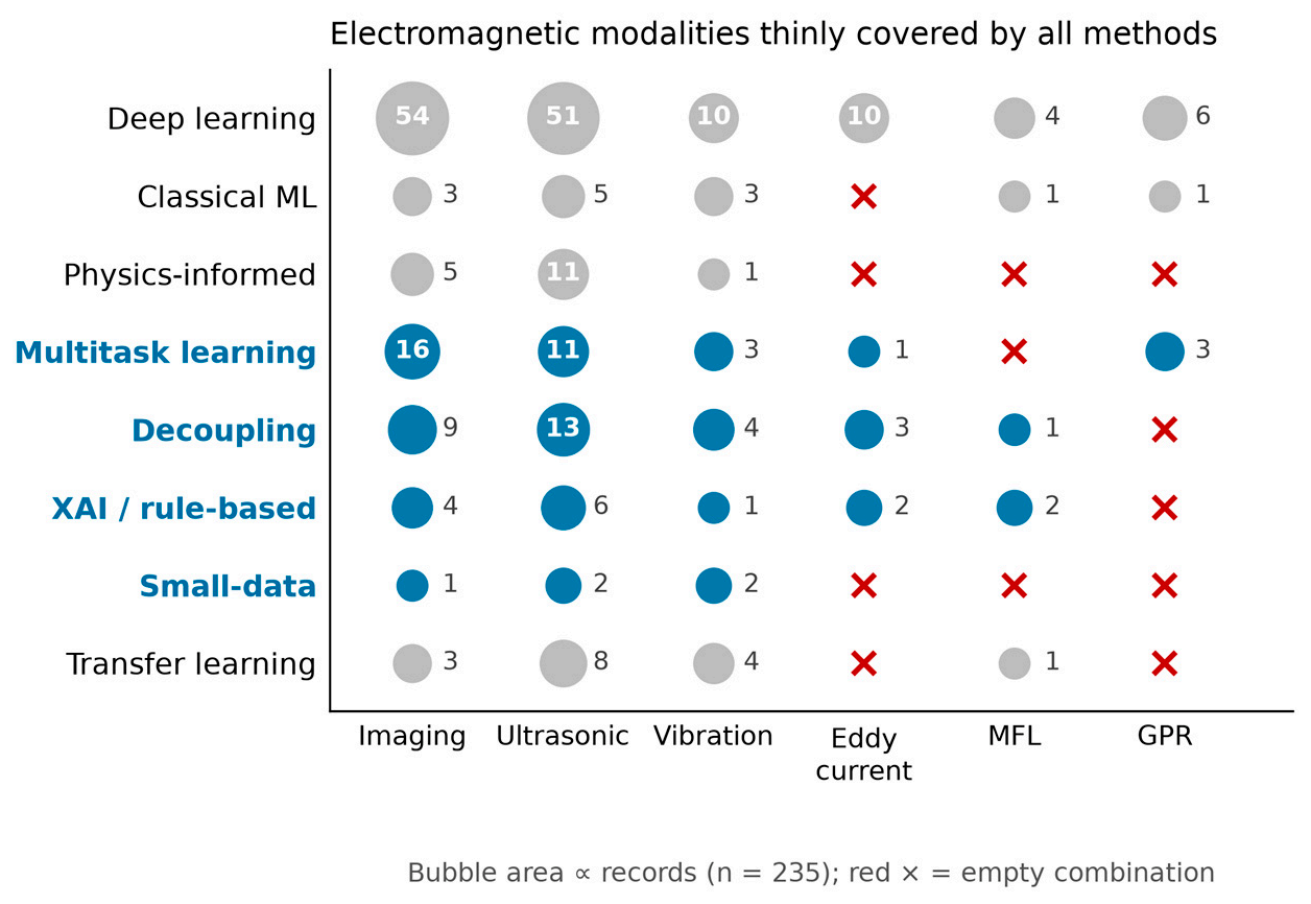
Method × modality landscape of the 235-record corpus. Bubble area is proportional to the number of records containing both concepts; a red cross marks an empty combination. Method families central to this work are shown in blue and bold. Concept membership was assigned by automated pattern matching over title, abstract, and both keyword fields.

Whereas most AI-NDT reviews focus on image-based inspection, the domain addressed here (one-dimensional electromagnetic signals) calls for a distinct set of methods: classical learners with handcrafted features for data-scarce settings [26,27], deep architectures for pipeline MFL sizing [26], and physics-informed formulations that reduce data needs via forward-model embedding [28]. Parameter estimation from electromagnetic signals is inherently coupled: permeability, conductivity, thickness, and lift-off often require decoupling for reliable estimates [29,30]. Analyzing 235 publications on AI for non-destructive evaluation (NDE) of reinforced structures on the method–modality grid (Figure 2) reveals uneven coverage: imaging and ultrasonics are well represented across methods, while MFL and eddy-current signals are mostly addressed by deep learning, despite mature instrumentation [31], leaving some combinations empty. This mismatch between industry needs and research is clear: the rise of deep learning has shifted focus away from the interpretable, physically grounded methods which are crucial in engineering. Meanwhile, XAI, which helps engineers understand, trust, and relate signal changes to physical phenomena, remains underutilized.

**Table 1:** Machine-learning method families in NDT by input-data modality.

Modality	Method Family	NDT Task	Key Strength and Limitation	Refs.
Images	Convolutional Neural Network (CNN)	Crack, spalling, corrosion detection	Potentially high accuracy; requires large labeled datasets	[21,32]
Images	U-Net, You Only Look Once (YOLO)	Segmentation; reinforcement detection in ground-penetrating radar (GPR)	Supports real-time inference; susceptible to the simulation-to-reality gap	[33,34]
1D electro- magnetic	Support Vector Machine (SVM), Support Vector Regression (SVR)	Defect sizing from handcrafted features	Suitable for small datasets; requires handcrafted features	[26]
1D electro- magnetic	Gaussian mixture models, logistic regression	Corrosion-severity classification from eddy-current features	Probabilistic, interpretable output; assumes class-conditional Gaussianity	[27]
1D electro- magnetic	Random forest, XGBoost	Regression of geometric parameters	Robust prediction; feature-importance estimates may be unstable at small *n*	[35,36]
1D elastic	1D-CNN, Long Short-Term Memory (LSTM)	Waveform classification; forecasting	Captures temporal structure; limited interpretability	[37]
1D (any)	Autoencoder	Anomaly detection; sensor fusion	Can be trained without labels; latent representation may be difficult to interpret	[38]
1D (any)	Physics-informed NN	Crack quantification from ultrasound	Lower data requirements; requires a forward model	[28]
Features	Principal Component Analysis (PCA)	Feature reduction	Computationally efficient; limited to linear transformations	[17]
Features	Sparse Bayesian learning	Multitask SHM; uncertainty-aware regression	Quantifies uncertainty; depends on prior specification	[7]
Features	Association rules, rule ensembles	Transparent feature–parameter relations	Interpretable by design; requires discretization	[23,39,40,41]
Decisions	Reinforcement learning	Inspection-path optimization	Supports sequential decision-making; often sample-inefficient	[26]

#### 1.2.3. Applications in NDT of Reinforced Concrete

Defect detection primarily uses deep CNNs to identify and grade cracks, spalling, and reinforcement corrosion from visual and thermographic images, with architectures now distinguishing reinforcement damage from the background in RC beams [42]. Structural health monitoring (SHM) uses vibration, strain, and acoustic data in LSTM forecasting models and autoencoder-based anomaly detectors, which are integrated into neural-network-based digital twins for RC bridges. These digital twins combine crack features with static and dynamic responses, to evaluate service performance [22,43]. Data-fusion techniques such as impact-echo, ultrasonic testing, and GPR enhance defect detection and bridge-deck mapping. Meanwhile, statistical fusion improves predictions of concrete properties [44]. This need for fusion reflects the complexity of RC inspection and highlights the difficulty of interpreting black-box decisions in multi-source settings.

A study-by-study survey of these approaches is given in Supplementary Section S7 (Table S3). First, no study recovers three coupled RC parameters simultaneously; most recover only two [33,37,45], showing the difficulty of multi-parameter identification. Second, when coupling is addressed, it is in a learned model or latent space, rather than through human-readable attributes [29,30], making results hard to audit, especially with small datasets and many factors. Although isolated studies address these issues [17,23], they are underrepresented, reflecting a broader lack of interpretable, data-efficient methods in this field.

#### 1.2.4. Challenges

Six interlocking challenges limit AI-based NDT adoption in RC. Table 2 lists each challenge and its engineering consequence; the corresponding coverage in the reviewed corpus is provided in the Supplementary Materials (Figure S10).

Table 2 identifies six challenges to AI adoption in NDT; this work focuses on the first three (data scarcity [24,46], interpretability [16,25], and parameter interdependence [29,30,37]) while the remaining barriers (simulation-to-reality gap [28,36,49], lack of standards [3,4], and measurement uncertainty [2,7]) provide broader context. Data scarcity limits applications to few-shot learning; interpretability is crucial for safety-critical assessments, where post hoc explainers such as SHAP [47,48] can be unstable, favoring transparent-by-design methods; and parameter interdependence causes models to capture cross-parameter correlations, rather than isolated effects, motivating decoupling. These issues underpin the approach discussed in Section 2.

### 1.3. Research Questions and Contributions

Section 1.1 and Section 1.2 identify three AI challenges in reinforced concrete: parameter coupling in MFL signals [29,30,37], data scarcity [24,46], and limited interpretability [16,25]. Table 3 maps these to the research questions (RQs). The main question is whether multiple structural parameters can be reliably and interpretably identified from sparse multilayer MFL data and limited measurements. Rather than maximizing accuracy, this work tests whether interpretable features can be linked to individual parameters, despite noise and low resolution. Section 2.1 discusses the main alternative, multitask learning (MTL), and compares it with the proposed method in Table 4.

This is a methodological study. Reinforced-concrete parameter identification serves as a case study because it combines three types of estimation problems in one pipeline: a discrete, rule-derived quantity (steel class), a discrete quantity resolved through sensor geometry (rebar diameter), and a continuous, curve-fitted quantity (cover thickness). Each requires a distinct evaluation tool. In response, this work extends our previous contributions [17,23,41] with the following:

1. A systematic literature review that identifies the key challenges limiting AI adoption in NDT and confirms the research gap;
2. A three-layer sensor array (5 mm inter-layer spacing) that extends ACO-based decoupling with geometric, inter-layer relationships, enabling diameter and cover-thickness estimation from features outside the ACO attribute set, alongside a statistically motivated sensitivity threshold selected using a conservative multiplier (sens.min, *k* = 4, ≈98.8% confidence), a genetic-algorithm fitting procedure for sub-sample peak localization, and statistically grounded validation of the pipeline’s key assumptions using only 20 configurations (400 measurements);
3. A dual evaluation framework matched to data type: rough-set confidence bounds (α_B_(*X*), γ_B_(*P*), boundary region BN_B_(*X*)) for discrete, rule-derived cases, and overlap-coefficient/error-propagation analysis for geometry- and curve-derived cases—demonstrating that the same pipeline supports rigorous, interpretable evaluation, regardless of whether the target is categorical or continuous;
4. A conceptual qualitative comparison against multitask-learning approaches, with quantitative comparison deferred to future work.

## 2. Related Works

This section reviews related work and the current state of the art related to the research questions, divided into two parts. Section 2.1 compares three common strategies for resolving coupled parameters (shared-representation multitask learning, latent disentanglement, and feature-level decoupling), and positions the proposed approach. Section 2.2 presents a systematic literature review (SLR) that maps trends and identifies research gaps in AI-based NDT of reinforced concrete.

### 2.1. Parameter Decoupling in the Context of Multitask Identification

Multitask learning (MTL) and disentangled representation learning represent two established approaches for handling multiple tasks or generative factors. MTL learns a shared representation across tasks [52], whereas disentanglement aims to isolate independent factors of variation in a latent space [50,51,56]. However, both paradigms present limitations particularly relevant to reinforced-concrete (RC) inspection: MTL requires extensive datasets and remains opaque, while disentanglement demands strong inductive biases and labeled probes to interpret its latent axes [54,55].

**Figure 3:**
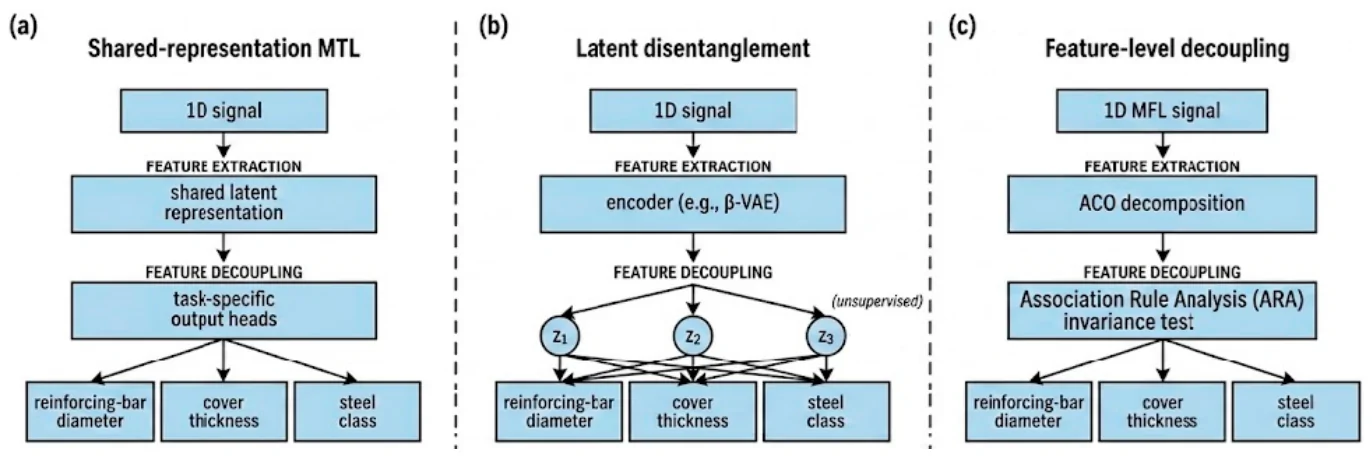
Three strategies for resolving three coupled reinforced-concrete parameters (reinforcing-bar diameter, cover thickness, and steel class) from a single 1D signal: (**a**) shared-representation multitask learning; (**b**) latent disentanglement; (**c**) feature-level decoupling proposed in this work.

By contrast, the feature-level decoupling proposed in this work operates directly on interpretable signal attributes derived from measurement physics. Table 4 and Figure 3 summarize and compare these three strategies across key constraints inherent to RC infrastructure inspection.

**Table 4:** Three strategies for resolving several coupled parameters from one measurement, compared against the constraints of reinforced-concrete inspection.

Criterion	Shared-Representation MTL	Latent Disentanglement	Feature-Level Decoupling (This Work)
Where separation occurs	Shared latent space	Latent axes of a generative model	Interpretable signal attributes
Supervision needed	Labels for every task	Labels even for model selection	A few reference measurements
Theoretical limitation/ verification mechanism	Negative transfer between tasks	Not achievable without inductive bias	Invariance verified per rule
Interpretability	Post hoc explanation	Latent axes are not inherently aligned with named physical quantities	Transparent by design
Error behavior	Errors may propagate across tasks	Residual entanglement may propagate errors	Designed to confine errors to a single parameter
Data demand	Typically high	Typically high	Lower
Key references	[6,7,52,53,57]	[15,50,51,54,55,56]	[17,23,41]

The comparison is conceptual, and is not based on matched experiments on the same dataset.

As shown in Figure 3a, multitask learning (MTL) retrieves parameters from a shared latent space that is not grounded in physical measurement, causing cross-parameter interference in which signals sensitive to one parameter are affected by others. MTL requires large datasets and acts as a black box. It often needs gradient-mitigation techniques such as gradient surgery to prevent task interference [53].

Latent disentanglement (Figure 3b) aims to separate generative factors using models like β-VAE [50,51,56]. However, its latent axes do not naturally align with physical quantities. Residual entanglement often remains, requiring labeled probes for interpretation and careful bias design [54,55], which is impractical under label scarcity.

Conversely, the proposed feature-level decoupling (Figure 3c) leverages ACO decomposition and Association Rule Analysis [23,41] to map one interpretable signal attribute to each specific parameter, based directly on measurement physics. This explicitly minimizes cross-parameter interference and eliminates the need for labeled probes or negative-transfer mitigations. Its data demand is limited to a few reference measurements, a scale below that at which MTL is documented, to operate effectively in the reviewed literature (Section 2.2).

To further enhance reliability, a subsequent rough-set analysis quantifies the confidence of each rule, clearly distinguishing definite from uncertain parameter ranges [58]. By establishing transparent feature–parameter relationships, this approach provides readable rules, directly addresses data-availability constraints in field applications [3,4], and serves as an effective calibration instrument for numerical models and digital twins [22,59].

### 2.2. Trends and Research Gap in AI-Based NDT of Reinforced Concrete

The research gap was delimited empirically through a systematic literature review (SLR) of Scopus-indexed studies, semantically screened using EmbedSLR, an open-source deterministic framework for embedding-based screening and bibliometric validation [1]. The search identified 235 records published after 2004; the 30 records semantically closest to the research question were retained, of which 22 met full-text eligibility criteria. The complete protocol (including the search string, research-question statement, inclusion criteria, PRISMA flow diagram, shortlist validation, and full bibliometric analysis of the corpus) is provided in Supplementary Materials, Sections S1–S5. Findings directly relevant to this contribution are summarized below.

Four findings characterize the field. First, its core concepts are recent, and growing rapidly: no record addresses multitask learning before 2015, yet in 2025–2026, multitask learning, explicit decoupling, and explainable or rule-based approaches appear in 24.7%, 20.8%, and 15.6% of records per period, respectively, a roughly tenfold increase in share over the preceding four years (Table S2). Second, modality coverage is uneven: all method families address imaging and ultrasonics, while deep learning handles nearly all magnetic flux leakage and eddy-current signals (Figure 2); notably, NDT-specific and simulation-focused records are under-represented in the shortlist, precisely the areas this work combines (Figure S4). Third, the community working on this intersection is fragmented: 88% of authors contribute only one record, and no single journal publishes more than 4% of the corpus, suggesting an unexplored niche, not a saturated field (Figures S5 and S9). Fourth, the gap is quantified: the most represented remedy for each of Table 2’s six challenges appears in no more than four reinforced-concrete records (Figure S10). The shortlist itself is validated by independent bibliometric indices: it shares 13.0× more references per pair than the full corpus (*p* = 0.014), confirming topical coherence (Table S1). The full bibliometric analysis (shortlist validation, temporal dynamics, geographic and journal distribution, keyword co-occurrence, and author-productivity patterns) is provided in Supplementary Materials, Sections S2–S5 (Figures S2–S9, Table S1).

The cross-tabulating method and domain tags locate the gap (Table 5). Multitask learning appears with reinforced concrete in six records, yet never with reinforcing-bar diameter, cover thickness, or magnetic flux leakage. Small-data and few-shot learning co-occur with none of the electromagnetic modalities or reinforcement parameters. Explicit decoupling appears with reinforced concrete in three records and with an electromagnetic modality in four, but with both in only one (precisely the intersection this work addresses).

Full-text screening confirms this gap: of the 22 eligible records, 16 address reinforced concrete. Among these, eight use a 1D electromagnetic modality, five explicitly decouple parameters, and only two simultaneously recover at least three (both from the present research group [23,41]). The closest independent study jointly estimates two coupled parameters, reinforcing-bar diameter and cover thickness, by inverting a dual-phase pulsed-eddy-current model with a two-stage network [37], consistent with earlier two-parameter GPR/electromagnetic induction (EMI) schemes [33,45] and radar/neural-network inversions [60,61]. Studies decoupling at least three quantities use ultrasound, not magnetic methods, and focus on damage, rather than reinforcement geometry [62]. This work fills a specific gap: transparent-by-design, feature-level, data-efficient decoupling of three coupled reinforced-concrete parameters (reinforcing-bar diameter, cover thickness, and steel class) from 1D magnetic signals.

## 3. Materials and Methods

This section outlines the experimental setup, measurement protocol, and signal-processing pipeline developed to decouple reinforced-concrete parameters from MFL data. The pipeline modifies the Cross-Industry Standard Process for Data Mining (CRISP-DM) for NDT by embedding data-scarcity mechanisms at every stage. Its eight stages are not project-management steps, but a sequence of data-scarcity methods, each extracting information otherwise requiring more specimens, scans, or labels. Each section below explains one stage and its contribution to data efficiency.

### 3.1. Experimental and Signal-Processing Protocol

**Figure 4:**
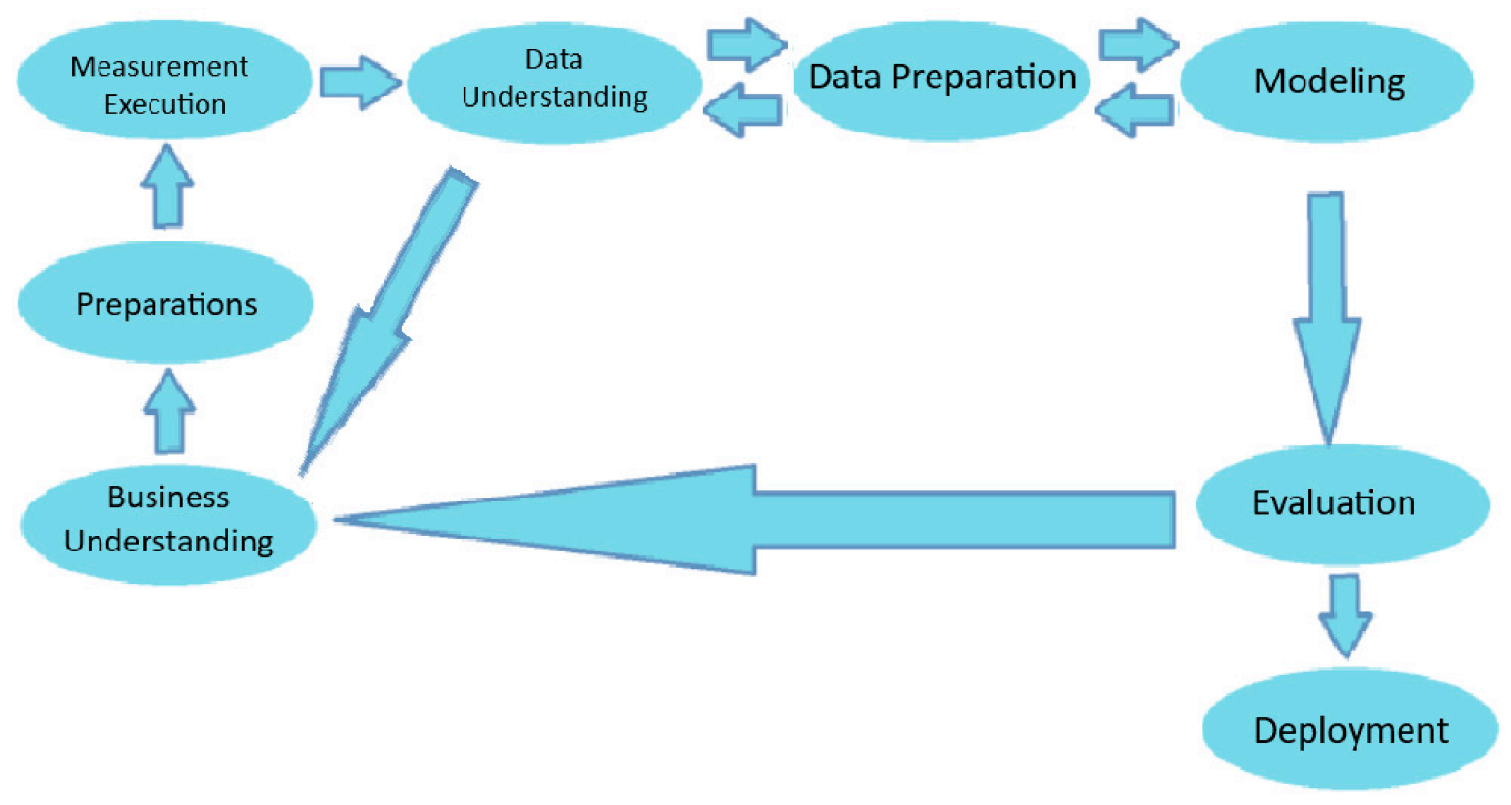
Model cross-industry standard process for data mining in NDT [41].

Unlike standard CRISP-DM, this pipeline treats physical measurement and processing as a first-class stage [41], and organizes its eight stages as a data-scarcity sequence, since measurement design determines what sparse-data NDT can infer. Figure 4 shows the stages and feedback paths; each stage’s data-efficiency mechanism is described below.

The *Business Understanding* stage defined the problem as separating the effects of coupled parameters (rebar diameter, concrete cover thickness, and steel class) from the MFL signal. A literature review using EmbedSLR [1] confirmed the research gap and identified three challenges: data scarcity, which limits every downstream stage; parameter interdependence, which prevents single-parameter inference; and limited black-box model interpretability, which undermines trust in safety-critical decisions. Of the six challenges in Table 2, no remedy appears in more than four reinforced-concrete records (Figure S10). Process simulations [63] supplemented the review, offering a controlled reference to interpret signal changes and test initial rule hypotheses, without physical specimens, before data collection. Supplementary Materials Section S1 details the review protocol, including the database, verbatim Scopus query, semantic-ranking research question, inclusion criteria, and PRISMA 2020 flow diagram (Figure S1).

The *Preparation* stage iteratively optimized the measurement setup, building on previous studies. It tested methods [41,64], configurations, magnetization schemes [63,64], and transducer types [23,41], while systematically tuning parameters for reliable results. It also calibrated devices, assessed limits such as maximum concrete cover and interference from adjacent rebars in dense meshes [63,64], and defined operating boundaries. Continuous feedback between hardware and signal quality ensured that each configuration was chosen to maximize information per specimen, an iterative strategy that reduces the number of reference measurements required.

The *Measurement Execution* stage scanned the MFL transducer perpendicular to the rebar, recording cross-sectional variations of all three magnetic-induction components. This 2D acquisition [23] provided spatial and directional information for feature extraction and decoupling of all three parameters in one scan, eliminating the need for separate measurement campaigns. Each campaign, however, is time-intensive, and unknown material properties and environmental variability make fieldwork unpredictable, leaving large labeled databases impractical (Section 3.2.1).

The *Data Understanding* stage uses Association Rule Analysis (ARA) [14] to connect signals to simulations, then to physical phenomena [41]. This comparison is what allows equal-interval feature extraction to reveal which waveform attributes respond to which parameter: a change observed in the measured signal can be matched against the corresponding simulated response, isolating the physical cause behind each attribute. ARA efficiently handles high-dimensional inputs by evaluating single-parameter pairs. However, strongly correlated equal-interval features generate overlapping, repetitive rules, which hinder interpretation, not computation. ACO decomposition [17] reduces each waveform to three uncorrelated attributes (amplitude, correlation, and offset), and its similarity-to-reference mechanism requires only a single reference waveform, so each rule offers distinct information. Visualizations and statistical summaries validate and select rules. This iterative process can reveal methodological, design, or measurement errors, prompting revisions to *Business Understanding* and ensuring later stages rely on accurate, meaningful data.

The *Data Preparation* stage identified interpretable signal features through ACO decomposition [17], each tied to a specific physical feature. This reduces parameter interdependence and cross-parameter interference before modeling. Preprocessing combined outlier removal, normalization, and filtering. Earlier equal-interval feature extraction with normalization was easy to implement and analyze, but produced many attributes, complicating models and weakening classifier performance through the curse of dimensionality [65]. The streamlined ACO approach limits features to nine per sensor (three per magnetic-induction component), improving RC-parameter decoupling and identifiability. This retains interpretability, while reducing overfitting risk under sparse data. A feedback loop to *Data Understanding* supports iterative signal-processing improvements. For example, rebar-diameter detection improved by filtering signals first, then replacing filtering with an approximation, and optimizing coefficients using a genetic algorithm [18]. This enabled sub-sample peak localization (0.1 mm from 1 mm sampling, effectively increasing resolution without additional measurements) and use of inter-layer relationships: displacement among peaks from three sensor layers estimates diameter invariant with respect to cover thickness across the tested range, while normalized inter-layer amplitude ratios estimate cover-thickness invariant with respect to diameter and steel class, across the tested range.

The *Modeling* stage applies Rough-Set Theory to analyze uncertainty in association rules from *Data Understanding*. Lower and upper approximations [58] separate definite from possible cases, revealing uncertainty zones and parameter ranges for reliable inference or refinement. Approximation accuracy *α_B_*(*X*) and classification quality *γ_B_*(*P*) quantify uncertainty, while the boundary region BN*_B_*(*X*) identifies ambiguous intervals. Unlike statistical validation, rough-set analysis provides a deterministic, distribution-free assessment of rule certainty, well suited to sparse data. This explicit approach improves ARA interpretability and parameter reliability, vital for safety engineering.

The *Evaluation* stage validates the pipeline for RQ1–RQ4 (Table 3), using rough-set measures and complementary tools. RQ1: The overlap coefficient (OVL) measures separation between target and non-target attribute distributions. RQ2: Rough-set approximations assess rule strength. RQ3: Decoupling without a shared latent space is verified by *α_B_*(*X*) = 1 across each parameter’s full range, with OVL supporting separation. For continuous cover thickness, RMSE and error propagation supplement these bounds. RQ4: Section 1.1 and Section 2.1 present the qualitative MTL comparison (Table 4, Figure 3). The evaluation adds empirical evidence: BN*_B_*(*X*) signals uncertainty, while statistical tests quantify trends and distributional differences across processing stages. Unlike standard protocols requiring large held-out validation sets, rough-set metrics use available measurements without additional labeled data.

The *Deployment* stage remains at technology readiness level (TRL) 4: validation is limited to laboratory specimens, with no testing on in-service structures or integration into portable field instruments (TRL 5+). Progress requires testing more concrete mixes, reinforcement configurations, and environmental conditions, and embedding association-rule inference in on-site hardware [65,66]. The pipeline’s explicit feature–parameter relationships and rough-set rule-certainty assessment are, in principle, suited to such integration and to calibrating numerical models and digital twins. Because rules are refined incrementally with each reference configuration, rather than retrained, field deployment avoids the large labeled datasets retraining requires. The Limitations Section discusses these aspects in more detail.

### 3.2. Preparations: Specimens and Measurement System

This section outlines the experimental setup, measurement protocol, and signal-processing pipeline developed to decouple reinforced-concrete parameters at the feature level, from MFL data. As this is a methodological study, the components are described in sufficient detail to ensure transparency, replicability, and adaptability by other researchers working with sparse NDT data.

#### 3.2.1. Samples

The RC specimens were designed to span the three target parameters:

- Reinforcing-bar diameter (*D*);
- Concrete cover thickness (*h*);
- Reinforced-steel alloy (*class*).

Diameter determines the steel cross-sectional area, and thus the reinforcement’s tensile-load capacity, directly governing the member’s structural capacity. Cover thickness protects the reinforcement against corrosion and fire, governs the structure’s durability and service life, and affects bond strength between steel and concrete.

Reinforcing steel is classified by yield strength (*Fyk*) and ductility [67]. Table 6 summarizes the three types examined in this study. Strength and ductility do not increase together: despite substantial differences in yield strength, A-I and A-III bars retain high ductility, with elongation at maximum force (*Euk*) > 5%. By contrast, the highest-strength class, A-IIIN, trades some ductility for greater load capacity. The classes also differ in surface geometry. A-I bars are smooth, whereas A-III and A-IIIN have distinct rib patterns; the double-skew ribs on A-IIIN improve bonding with the surrounding concrete.

Ductility allows a reinforced-concrete member to form a plastic hinge and redistribute bending moments before failure, providing warning deformation, rather than brittle rupture. Steel class is a safety parameter, not just a material spec: in existing structures with incomplete documentation (which motivates this work), an unknown steel class means an unknown ductility reserve, impacting safety under load. Different rib geometries across classes plausibly influence the steel class’s effect on the measured MFL signal, aligning with the feature attributes (amplitude and offset) used for its identification.

The specimens were designed to span ranges representative of typical construction practice. Cover thickness varied from 20 to 60 mm in 10 mm increments, exceeding the 20–50 mm range most commonly encountered in RC structures [68]. Two rebar diameters (10 mm and 12 mm) and three steel classes (A-I, A-III, and A-IIIN) were tested. The resulting specimen set (Table 7) intentionally omits some diameter–class combinations, since the pipeline is designed to identify each parameter from a small number of reference measurements, rather than an exhaustive factorial design.

This design includes 20 physical specimen configurations spanning four diameter–class combinations and five cover-thickness levels, with each configuration scanned 20 times. Sampling only part of the parameter space reflects practical NDT constraints: preparing even one specimen requires precise formwork, controlled curing, accurate rebar placement, and repeated scans under stable conditions. Preparing all 20 configurations takes weeks of laboratory work, and uses substantial space. The final measurement campaign (20 scans per configuration, 96 points per scan, and a 0.5 s settling time) took several days, while iterative recalibration extended the overall process to more than a month. Extending the study to every *D*–*h*–class combination would require hundreds of specimens and years of work, making it infeasible in academic or industrial NDT. These constraints (specimen preparation, curing, parameter control, and multi-layer scanning) are resource-intensive, and limit most experimental NDT programs.

Data-efficient, physically grounded methods are essential in fields like NDT, where exhaustive datasets are rarely attainable. The dataset provides a basis for evaluating whether physically interpretable rules support interpolation within the tested range, while the 400 scans support assessment of the measurement pipeline, but enable calibration from few reference configurations.

Not every diameter–steel-class combination was cast. The four configurations (Table 7) include two steel classes per diameter and both diameters for A-IIIN, allowing each parameter’s effect to be assessed across at least two levels of the other. However, A-I with 12 mm bars and A-III with 10 mm bars were not cast, partially aliasing diameter and steel-class effects in these cells. Thus, the invariance reported in Section 4.4 and Section 4.5 is established only for tested combinations; this design cannot detect confounding limited to uncast cells. Still, because each feature is tied to a specific physical phenomenon, its response can be reasoned consistently across uncast cells and the intervals between tested configurations. The same reasoning extends beyond the tested range (for example, a predictable inter-layer peak-displacement shift from 10 to 12 mm suggests a similar shift from 12 to 14 mm), though both require experimental confirmation.

This physical interpretability makes the pipeline well suited to explainable AI in NDT: each rule links a named signal attribute to a physical mechanism, keeping feature-level reasoning transparent and allowing incremental expansion without black-box retraining. Few-shot learning calibrates these rules from limited reference measurements, extracting maximum information from minimal configurations and providing a transparent, extensible foundation for future data collection.

#### 3.2.2. Measurement System

**Figure 5:**
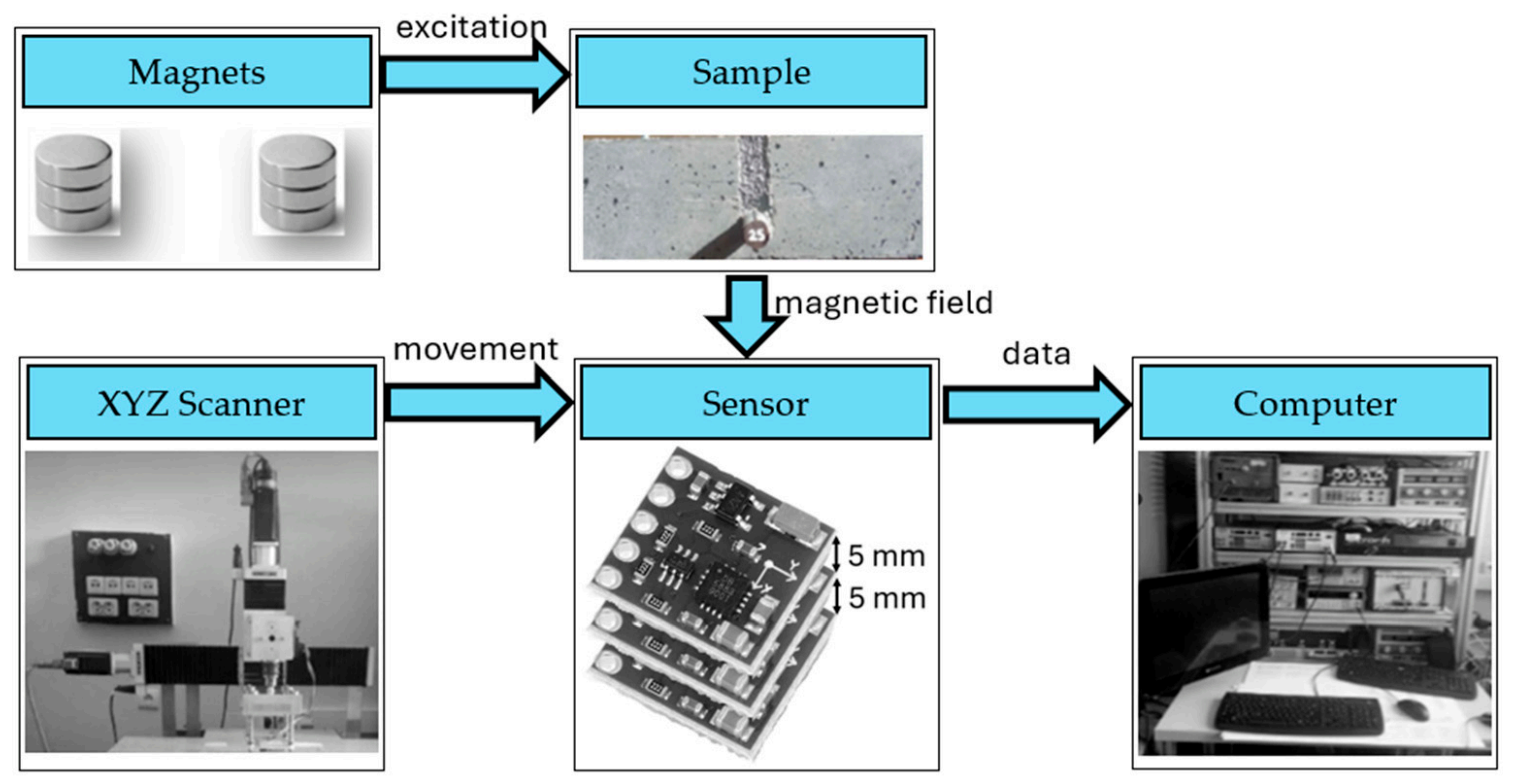
Block diagram of the measurement system, with the three-layer sensor board shown in the inset.

The measurement system (Figure 5) comprises four subsystems: excitation, positioning, three magnetic field sensors (HMC5883L, Honeywell, NJ, USA [69]) at lift-off distances spaced 5 mm apart, and data acquisition. Two stationary neodymium magnets above the rebar excite the specimen in a same-pole magnetization (SPM) configuration [63]. An XYZ scanner moves the sensors perpendicular to the rebar, while the magnets remain fixed. Each sensor records a 1D scan of the three spatial components of magnetic induction (*B*_x_, *B*_y_, *B_z_*) across the rebar; the three parallel scans form a 2D dataset for feature extraction. The data-acquisition subsystem sends signals to a computer for processing.

The three-layer arrangement enables decoupling and is therefore specified in full. The magnetometers operate at factory defaults: ±1.3 Gauss full scale, 0.92 mG/LSB resolution, and 15 Hz output. At each scan point, the stage dwells for 0.5 s and averages readings, reducing per-point noise below the single-sample noise floor. The sensors are mounted coaxially at 0, 5, and 10 mm above the specimen. The 5 mm spacing matches the cover-thickness step, so leakage-flux peak displacement between adjacent layers reproduces that between adjacent cover thicknesses, enabling the cover-independent diameter estimate in Section 4.4.1. Smaller spacing would put this displacement below the genetic-algorithm fit’s 0.2 mm peak-localization precision; larger spacing would place the outer sensor in the low-SNR region described in Section 4.2.

**Figure 6:**
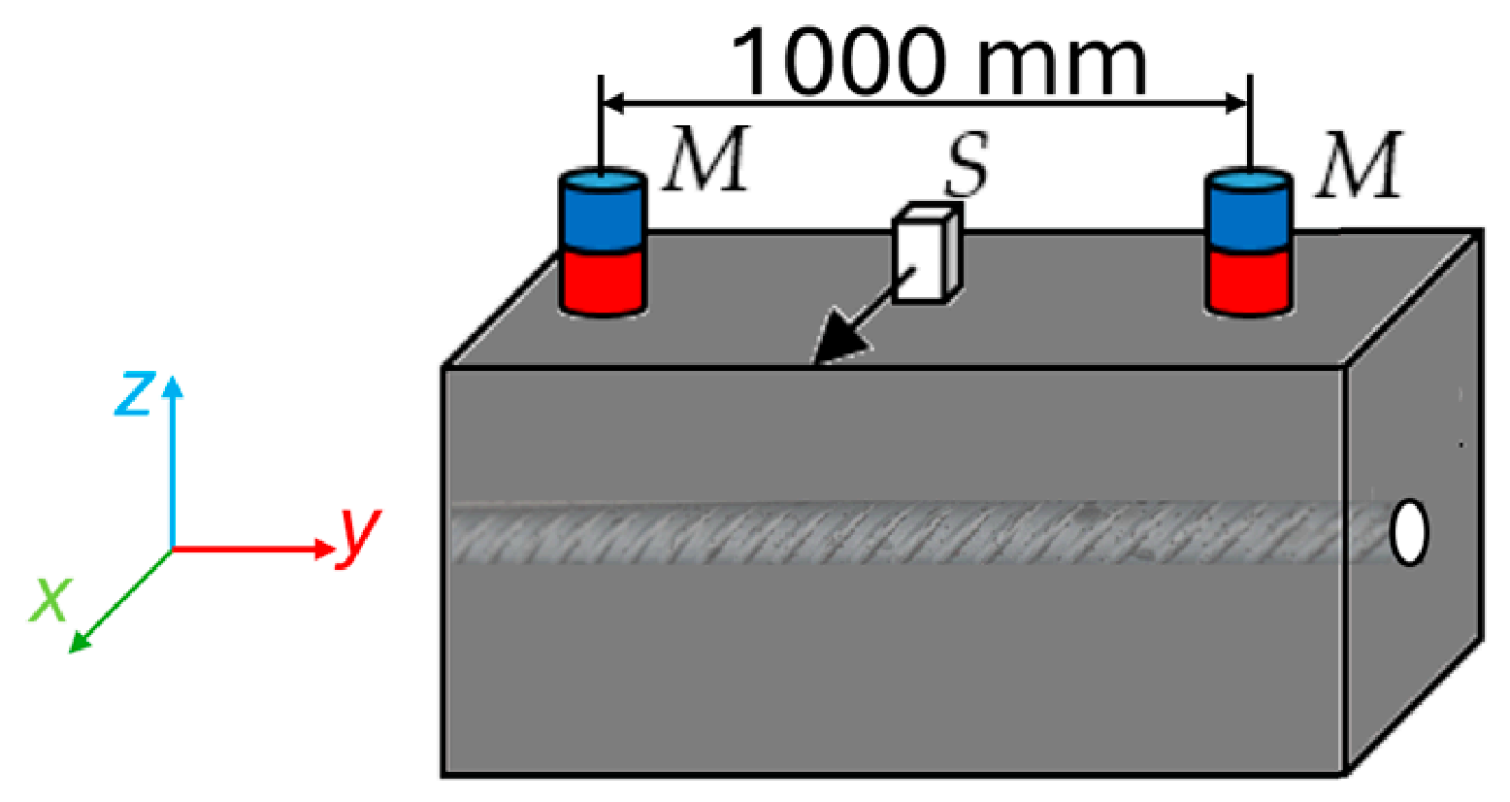
Measurement setup and coordinate system: two stationary magnets (M) are placed on the specimen surface above the rebar. The sensor array (S) scans perpendicular to the rebar, along the X axis. Y runs along the rebar, and Z is the vertical lift-off.

Excitation uses two stationary NdFeB permanent magnets (grade N42, NiCuNi-coated) positioned above the rebar in a same-pole configuration [63]. Each pole comprises two stacked discs with a nominal diameter of 55 mm and a height of 10 mm each, for a total height of 20 mm. The poles are 1000 mm apart along the bar axis (Figure 6), approximately 500 mm from the sensor array, which scans the gap. Since the magnets remain stationary as the array moves, the magnetizing field at each specimen point stays constant during scanning; thus, inter-layer amplitude ratios depend only on sensor-to-rebar distance. The XYZ stage advances in commanded 1 mm steps; its much finer mechanical repeatability is negligible, compared with the 1 mm spatial quantization discussed in Section 3.5.

### 3.3. Measurement Execution: Acquisition Protocol

The protocol standardized-sensor translation perpendicular to the rebar over 96 mm at 1 mm intervals, with the stage pausing 0.5 s at each point to settle; positioning repeatability was well below the 1 mm step and negligible versus spatial quantization. The scan was intentionally asymmetric: the sensor started above the rebar at 36 mm, and advanced 60 additional 1 mm steps. Based on waveform symmetry observed in preliminary studies [17,41], this reduces measurement time without losing signal information. Perfect symmetry is an idealization (magnet-force imbalance and imperfect rebar centering cause slight asymmetries, even in labs), and peak-based features are sensitive to them, so extending the scan range will not significantly reduce this variability.

At each lift-off level, all three magnetic-field components (*B*_x_, *B*_y_, and *B*_z_) were recorded simultaneously, and treated as separate waveforms [23]. Each setup was repeated 20 times for statistical robustness and repeatability. If alignment was suspected to deviate (most often at small cover thicknesses, where the sharper field profile is more sensitive), the specimen was rechecked against the reference alignment and remeasured, before the scan was accepted.

Experiments were conducted at 20–25 °C on non-metallic supports to prevent electromagnetic interference. Before each series, the system was calibrated with a reference specimen. Figure 6 shows the setup and coordinates: X is the scan direction perpendicular to the rebar, Y runs along it, and Z is vertical lift-off.

### 3.4. Data Understanding: Feature-Extraction and Association Rules Analysis

The initial phase of *Data Understanding* involved extracting signal features to reveal relationships between measured waveforms and the physical parameters of reinforced-concrete specimens.

**Figure 7:**
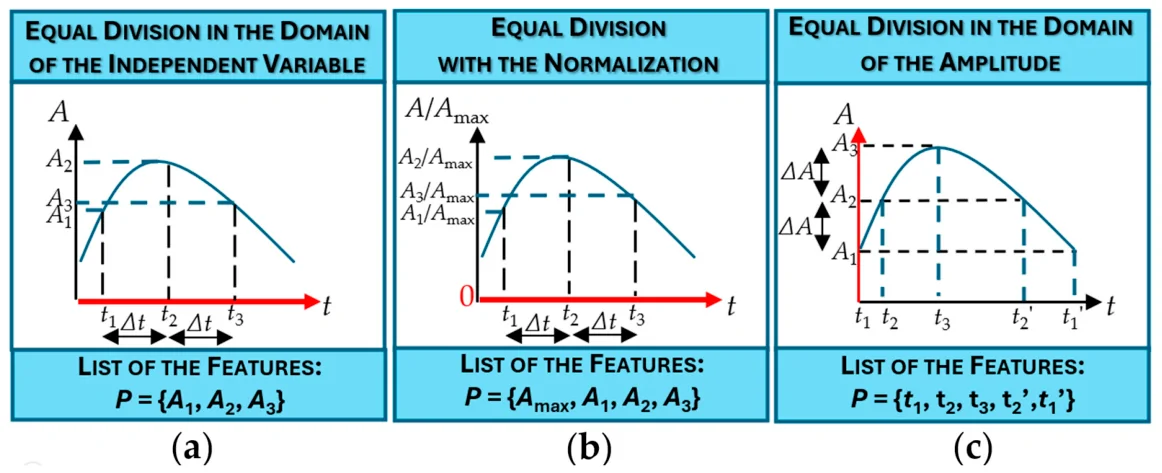
Feature Extraction Through Equal Division: (**a**) in the independent variable domain, (**b**) example with normalization, (**c**) in the amplitude domain.

Three approaches were employed: (I) equal intervals in the domain of the independent variable, which records amplitude at equally spaced scan positions, but requires many attributes; (II) equal intervals in the amplitude domain, increasing sampling density in regions of rapid change and improving shape representation, though it may cause ambiguity when one amplitude maps to multiple positions; and (III) equal intervals with normalization: in all three, accuracy improves by normalizing the waveform before shape-attribute extraction, decoupling amplitude from shape [14,17]. The article [17] describes the methods in detail. Figure 7 presents equal-division methods.

Equal-division methods are useful for exploratory analysis and association-rule discovery because they require no prior knowledge and are easy to implement. However, they generate numerous attributes, increasing the risk of overfitting and the curse of dimensionality [65]. Shape attributes are often correlated, violating principles of minimal attribute count and low correlation for accurate waveform-shape representation. These methods also demand extensive attribute reduction (which is time-consuming and subjective, with filtering or wrapper methods) and can disconnect features from real waveform parameters when PCA is used [14,17].

**Figure 8:**
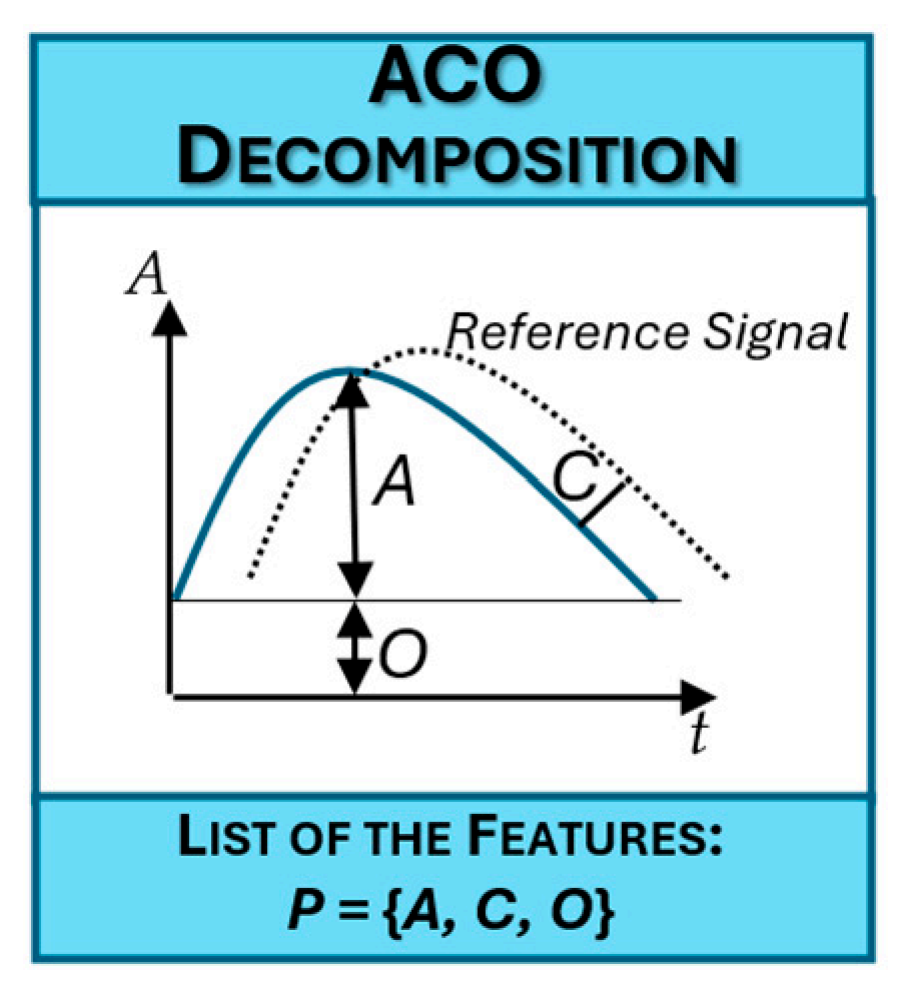
Feature extraction through ACO (Amplitude–Correlation–Offset) decomposition.

Initial investigations favored equal intervals for simplicity [17]. To overcome their limitations, the approach evolved into ACO decomposition [17]. ACO reduces waveforms to three uncorrelated attributes: amplitude (max–min), offset (the waveform’s minimum value, marking its vertical displacement from the zero baseline), and correlation (waveform shape against a reference signal). It can act as pattern recognition, comparing each measurement to a single reference waveform; this similarity-to-reference mechanism makes ACO inherently suited to few-shot settings, resolving correlated shape-attribute issues without a large rule database. This concise, uncorrelated feature set better decouples and identifies diameter, cover thickness, and steel class, while preserving interpretability and reducing overfitting [14,17]. In this work, ACO is treated as part of Data Understanding, though it also belongs to Data Preparation, particularly in selecting the attribute-extraction method; its attributes form the basis for association-rule analysis, linking each attribute to the corresponding physical RC parameter. The method is detailed in [17] and illustrated schematically in Figure 8.

The rule database is built from all record pairs differing in only one structural parameter *p* (the smallest available change), following prior work [41]. Repeated scans remain separate records, but are treated as clustered observations within each specimen. Each one-parameter configuration pair contributes 20 × 20 = 400 record pairs, included without averaging, preserving the full measurement-noise distribution in the sensitivity-threshold filtering (1) and rough-set analysis.

Structural parameters are organized into discrete series (e.g., standard rebar diameters and steel classes), so the smallest meaningful change is clearly defined. The measured signal, by contrast, is continuous: two records that are nominally identical still differ slightly, and these differences come from noise, rather than from an actual change in any parameter. Consequently, a small feature difference between two records cannot, by itself, be treated as evidence of a real parameter change. To prevent labeling such fluctuations as genuine changes, this paper introduces a sensitivity threshold (*sens.min*), unlike previous work [41]. Each record combines the three structural parameters with the nine ACO attributes extracted from the signal. For a pair of records differing only in one structural parameter *p* (e.g., diameter), denote the two values as *p*_low_ and *p*_high_. For each ACO feature (*f*) independently, let *f*_low_ and *f*_high_ be its values in the records corresponding to *p*_low_ and *p*_high_, respectively, and let Δ*f* = *f*_high_ − *f*_low_. The attribute is then labeled as (1):

↑, *if* Δ*f* > *sens*.*min*; (−), *if* |Δ*f*| ≤ *sens*.*min*; ↓, *if* Δ*f* < −*sens*.*min* (1)

Since noise levels vary with cover thickness [63], *sens.min* is treated as an adjustable parameter, rather than a fixed constant, allowing the threshold to match the measurement quality of each range. It is not optimized arbitrarily: too high a value suppresses genuine changes, too low a value mislabels noise as signal, and a stable plateau typically exists where noise is suppressed while true parameter-driven variations are preserved. Here, the threshold was set using a statistically motivated heuristic with a conservative multiplier, requiring only three or four reference measurements. With fewer records, estimates become less stable; the class space is divided equally, based on a deterministic few-shot calibration rule (see Section 4.3). This rule accounts for worst-case noise, small-sample correction, and pairwise-comparison inflation. This calibration constitutes a key methodological contribution of the present work. A rule then takes the general form (2):

If (*BODY*) Then (*HEAD*) [*supp*, *conf, sens.min*] (2)

where *BODY* (*A*) is the change in a physical parameter, and *HEAD* (*B*) is the corresponding *sens.min*-filtered change in an ACO attribute. Rule quality is characterized quantitatively by support (*supp*) and confidence (*conf*), and qualitatively by *sens.min*. Support is the proportion of database records (*D*) containing the rule’s pattern (3):

(3) $$\mathrm{supp}\;\left(A\right)=\frac{\#\{d_{i}\in D:A\subset d_{i}\}}{\#D}$$

When analyzing an association rule of the form *A → B*, where *A* and *B* are disjoint itemsets (*A* ∩ *B* = ∅), the support of the rule is the support of the combined itemset *A* ∪ *B*, which is described as (4):

*supp* (*A* → *B*) = *supp* (*A* ∪ *B*) (4)

Confidence is the conditional probability that the rule holds, given by (5):

(5) $$\mathrm{conf}\left(A\to B\right)=\;\frac{\mathrm{supp}\;\left(A\cup B\right)}{\mathrm{supp}\;\left(A\right)}\approx P(B|A)$$

As in prior applications, set *A* is restricted to the three physical parameters and set *B* to the ACO attributes, with the *BODY* length fixed to one element—substantially reducing the search space relative to classical Apriori [70].

### 3.5. Data Preparation: Genetic-Algorithm Optimization for Noise Filtration

*Data Preparation* addressed two limitations identified during the *Data Understanding* phase: (I) measurement noise; and (II) 1 mm spatial quantization of the scan, both restricting precise waveform peak location from raw samples.

The problems are illustrated using signal-to-noise ratio (SNR), given as (6):

(6) $$\mathrm{SNR}=20\log_{10}\frac{A_{\mathrm{signal}}}{A_{\mathrm{noise}}}$$

Since the waveform peak often rests on a nearly flat plateau over several samples, noise can bias the apparent maximum. Noise reduction proceeded iteratively: a three-sample median filter first removed isolated outliers (sparse, high-amplitude deviations) without altering the waveform shape. A fourth-order low-pass Butterworth filter (Wn = 0.2, ~10 mm wavelength) was then tested to reduce noise further. Because causal Infinite Impulse Response (IIR) filters shift phase and bias peak position [71,72], and diameter and cover thickness are read from the peak position, a continuous model was used instead, to identify the peak analytically, avoiding such distortion.

The fitted model combines (sums) two derivative-of-Gaussian components for *B*_x_ and two Gaussian components for *B*_y_ and *B*_z_, each with an independent amplitude, mean, and standard deviation. This form was chosen based on empirical evidence, because it accurately captures the characteristic bipolar (peak–dip) shape observed in measured MFL signals, using a simple model that avoids asymmetry and overfitting [17,41]. Fitting this six-parameter, nonlinear model to raw data is more effective after outlier removal: although least-squares fitting reduces noise, it is vulnerable to large, sparse outliers that can skew results. A median filter is therefore applied before fitting.

The six-parameter, nonlinear fit is prone to local minima, a problem exacerbated by measurement noise and the model’s exchange symmetry (swapping the two components leaves the fit unchanged). Gradient-based least-squares methods are particularly vulnerable: they require a per-waveform initial guess, and noisy data makes numerical gradient estimation unstable, increasing the risk of spurious minima. A genetic algorithm (GA) was therefore used instead—a population-based, derivative-free global search that explores multiple candidate solutions in parallel, making it inherently more robust to local minima, requiring no initial guess, and naturally accommodating the model’s symmetry [18]. The GA evolved 100 candidates over 200 generations using tournament selection (size three), arithmetic crossover (rate 0.8), Gaussian mutation (10% per parameter), and 5% elitism, minimizing the mean squared error between the fitted curve and the measured waveform.

Peak positions at 0, 5, and 10 mm lift-off distances were extracted and used to determine diameter and cover thickness: across the tested range, inter-layer peak displacement Δ*x* estimates diameter with no measurable dependence on *h*, while the ratio of normalized peak amplitudes across layers estimates cover thickness with no measurable dependence on *D* or steel class.

**Figure 9:**
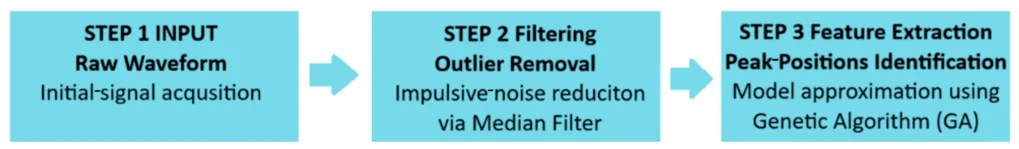
Noise-filtration and peak position-extraction process.

The noise-filtration and peak-extraction process is shown in Figure 9.

### 3.6. Modeling: Rough-Set Uncertainty Analysis

The *Modeling* stage quantifies the uncertainty inherent in association rules derived during *Data Understanding* and refined via GA-based curve fitting. Association rules identify qualitative relationships (e.g., “if cover thickness increases, then amplitude decreases”), but neither specify the precise parameter bounds over which a rule holds reliably nor quantify regions of ambiguity. In practice, a rule may hold over one segment of a parameter’s range but become unreliable over another, yet association rules alone cannot identify where this transition occurs (e.g., as cover thickness/lift-off increases, SNR decreases, affecting rule certainty). Rough-Set Theory [58] addresses this by modeling imprecise and uncertain knowledge through set approximations. From an XAI perspective, rough sets suit NDT by creating clear, deterministic decision boundaries that engineers can easily understand (unlike black-box models). They partition the measurement space into regions of certainty, potential, and ambiguity, providing interpretable, rule-based confidence measures for domain experts to review and validate (essential in safety-critical inspections [73]).

The rough-set analysis used all 400 measurements (20 specimen configurations × 20 repeated scans), with no training–test split; results are therefore descriptive and in-sample. Approximation accuracy *α*_B_(*X*) and classification quality *γ*_B_(*P*) measure decision-class overlap, not rule performance on new specimens; such measures offer limited value for comparing models [74]. Because reducts depend on the sample, dynamic reducts assess stability across random sub-tables [75]. In-sample rule strength fits this methodological aim: it shows whether rules hold across repeated measurements, which is the question RQ2 addresses. Stability analysis and external validation on new specimens remain the natural next steps (Section 5.3).

In the formalization of rough-set theory, let *U* be a non-empty finite set of measurement objects and *F* = {*f*_1_, *f*_2_, ..., *f*_9_} be the conditional attribute set representing the nine ACO attributes extracted from a single sensor (three per magnetic-induction component), treated separately for each of the three layers. Let *P* = {*p*_1_, *p*_2_, *p*_3_} denote the set of structural parameters (cover thickness, rebar diameter, and steel class). For a target decision class *X* ⊆ *U* representing a specific structural parameter state (i.e., a bounded range of a target parameter *p* ∈ *P*), the lower approximation $\underline{B}X$ is defined as (7):

(7) $$\underline{B}X=\left\{u\in U\;\right|\;{\left[u\right]}_{B}\subseteq X\}$$

When the upper approximation $\bar{B}X$, with respect to an attribute subset *B* ⊆ *F*, is defined as (8):

(8) $$\bar{B}X=\left\{u\in U\;\right|\;{\left[u\right]}_{B}\cap X\neq \emptyset \}$$

where [*u*]*_B_* denotes the equivalence class of an object *u* ∈ *U* determined by the discretized values of attributes in *B*. The lower approximation contains objects that certainly belong to the target class *X*, while the upper approximation contains objects that possibly belong to *X*. The difference between them constitutes the boundary region $BN_{B}\left(X\right)=\bar{B}X\setminus \underline{B}X,$ which explicitly isolates parameter intervals where membership is uncertain due to signal noise or cross-parameter interference.

**Figure 10:**
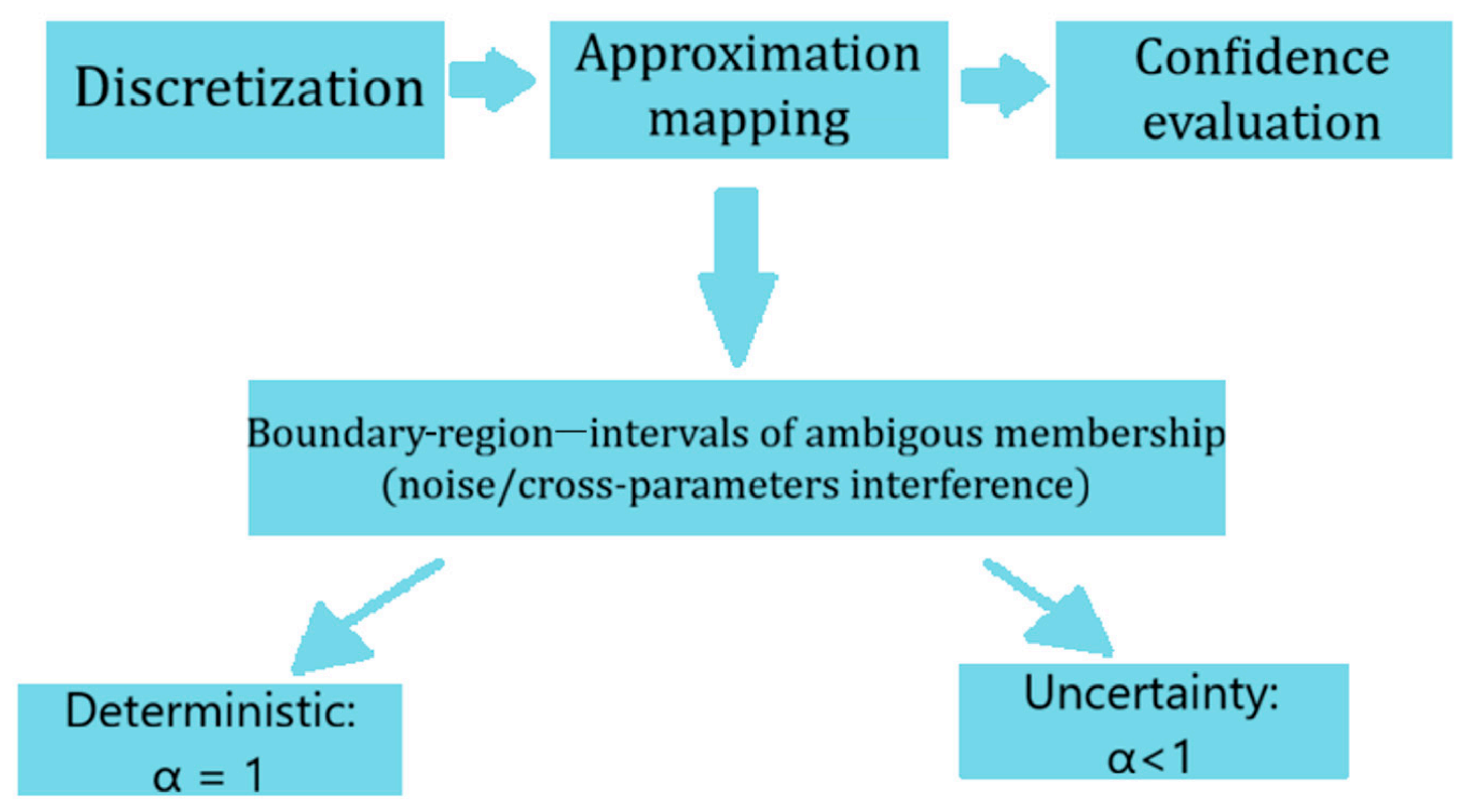
Rough-set uncertainty analysis.

The rough-set uncertainty analysis uses three steps (Figure 10). First, each continuous ACO attribute is discretized into interval labels based on Δ*f* = *f*_high_ − *f*_low_, using the adaptive sensitivity threshold (sens.min) from Section 3.5 to filter random noise between record pairs. Second, approximation mapping uses the lower approximation to identify ranges where an ACO attribute *f* responds unambiguously across repeated measurements for each structural parameter *p* ∈ *P*. The upper approximation captures all candidate ranges, highlighting regions needing additional measurements. Third, confidence is evaluated by calculating approximation accuracy *α_B_*(*X*), the ratio of cardinalities given in (9):

(9) $$\alpha_{B}(X)=\frac{\left|\underline{\underline{B}X}\right|}{\left|\bar{\bar{B}X}\right|}$$

where *α*_B_(*X*) = 1 indicates a fully deterministic, noise-invariant rule, and α_B_(X) < 1 measures boundary uncertainty. If *α* falls below this threshold, the rule is treated as unreliable, indicating that additional measurements are required [58].

While *α*_B_(*X*) quantifies the reliability of a single decision rule for a target class *X*, it does not capture the overall classification power of the attribute set *B* across all decision classes. To address this limitation, the classification quality γ_B_(*P*) is also computed as a global metric defined in classical rough-set theory [58] as (10):

(10) $$\gamma_{B}\left(P\right)=\frac{\left|\underset{X\in U/P}{⋃}\underline{B}X\right|}{\left|U\right|}$$

where *U*/*P* denotes the partition of the universe *U* into decision classes defined by the structural parameter *P* (e.g., cover-thickness ranges, rebar-diameter bins, or steel types). The numerator sums all lower approximation cardinalities across decision classes, while the denominator is the total number of objects. Intuitively, *γ_B_*(*P*) reflects the proportion of all measurement instances that can be unambiguously classified based solely on the conditional attributes *B*, without falling into boundary regions. While *α_B_*(*X*) provides rule-level confidence for a specific parameter state, *γ_B_*(*P*) provides dataset-level discriminative power, offering a complementary global evaluation metric [58].

### 3.7. Evaluation: Protocol

The rough-set formalism introduced above directly addresses the research questions formulated (Table 4). Regarding Feature Sensitivity and Invariance (RQ1), the lower approximation provides a strict, quantitative criterion identifying the tested parameter ranges over which a specific ACO attribute remains invariant to changes in the other structural parameters. To address Rule Derivation from Sparse Data (RQ2), replacing qualitative claims with the approximation accuracy *α_B_*(*X*) converts sparse empirical rules into bounded probabilistic confidence measures without requiring large-sample distributional assumptions. Furthermore, Decoupling Without Shared Latent Space (RQ3) is achieved by confirming that for each structural parameter *p* ∈ *P*, there exists an ACO attribute *f* ∈ *F* whose lower approximation covers the entire operational range of *p* while remaining invariant (*α_B_*(*X*) = 1) to variations in non-target parameters. This bypasses the negative transfer and latent entanglement inherent inmulti-task deep networks (Table 4, Figure 3c). Finally, Table 4 and Figure 3 address interpretability and noise robustness relative to MTL (RQ4) by qualitatively comparing the decoupling strategies. Unlike deep MTL feature-importance rankings that vary run-to-run, the rough-set boundary region BN*_B_*(*X*) provides a deterministic, transparent measure of model confidence. As measurement noise increases, the expansion of BN*_B_*(*X*) explicitly alerts the system operator to degraded data quality, supporting auditable decision-making [7].

The rough-set confidence bounds offer an operational feedback loop: they validate trustworthy prediction intervals and highlight where more measurements or sensor adjustments are needed. This supports the iterative NDT-focused Cross-Industry Standard CRISP-DM framework.

Feature separation and invariance (RQ1) are quantified, using the overlap coefficient (OVL) between the empirical distribution of each ACO attribute conditioned on its target parameter and its distribution conditioned on each of the two non-target parameters. For two probability density estimates *f* and *g*, the overlap coefficient is defined as (11):

(11) $$\mathrm{OVL}\left(f,g\right)=\int \min\left(f\left(x\right),g\left(x\right))\;\mathrm{dx}\right)$$

with values close to 0 indicating strong separation and values close to 1 indicating strong overlap [76]; a value of exactly 0 corresponds to fully non-overlapping distributions. The interpretation of OVL values and the classification of attribute–parameter relationships as “separated” or “overlapping” are based on the empirical distributions presented in Section 4, supported by visual and statistical evidence.

Rule derivation from sparse data (RQ2) is assessed using the accuracy of approximation *α_B_*(*X*) defined in (9) and the quality of classification *γ_B_*(*P*) defined in (10), reported per rule and per parameter, respectively. Unlike standard evaluation protocols [77] that rely on large validation splits and parametric assumptions, the rough-set metrics *α_B_*(*X*) and *γ_B_*(*P*) provide a deterministic, distribution-free assessment of rule certainty and global discriminative power directly from the available empirical bounds, making them inherently suited to the small-data setting of this study.

Since peak-position precision is physically relevant to *h* and *D*, an ablation comparing raw, median-filtered, and median-filtered + GA-fitted signals is performed for all three parameters. For the steel class, the same processing chain is used, but GA fitting is unlikely to improve results much, since steel-class data is encoded in amplitude and offset, not spatial peak position. Filtering and fitting offer robustness against outliers, and are kept.

Decoupling without a shared latent space (RQ3) is verified by checking, for each structural parameter *p* ∈ *P*, whether its assigned ACO attribute satisfies *α_B_*(*X*) = 1 with a lower approximation spanning the entire tested range of *p*, and by examining how the rough-set boundary region BN*_B_*(*X*) varies across the operating range identified in Section 4, linking regions of reduced reliability to the underlying SNR degradation.

Interpretability and robustness (RQ4) are discussed, based on Section 2.1 and Section 4. The deterministic rule-based method contrasts with MTL feature-importance rankings, which vary due to randomness [35]. The association rules and confidence measures (*α_B_*(*X*), *γ_B_*(*P*)) are fully deterministic on the same dataset. The rough-set boundary region BN*_B_*(*X*) indicates uncertainty, which depends on signal quality, as shown in Section 4. This built-in uncertainty signaling is not available in standard MTL predictions and is summarized in Table 4. Unlike black-box models that need explanation methods, the rough-set framework offers inherent interpretability: decision boundaries (lower/upper approximations) and confidence measures (α, γ) are derived directly from data, making evaluation transparent and auditable.

### 3.8. Deployment: Technology Readiness

The nine-level TRL scale [66] measures technology maturity from research to deployment. On the basis of laboratory testing, the technology described in this study is assessed at TRL 4. Here, TRL serves as an innovation-management tool, clarifying what the evidence does and does not support and informing go/no-go investment decisions [66].

The constraints that place the study at this level, and the path beyond it, are set out in Supplementary Section S8. The Limitations Section discusses detailed limitations and steps toward higher TRL levels.

## 4. Results

This section presents the experimental results processed through the pipeline described in Section 3. The results address the four research questions (Table 3), moving from raw-signal observations, through quantitative decoupling, to parameter-specific evaluation. All results derive from 20 specimen configurations and 400 measurements—a dataset size set by specimen-preparation constraints, not by choice.

### 4.1. Measurements

**Figure 11:**
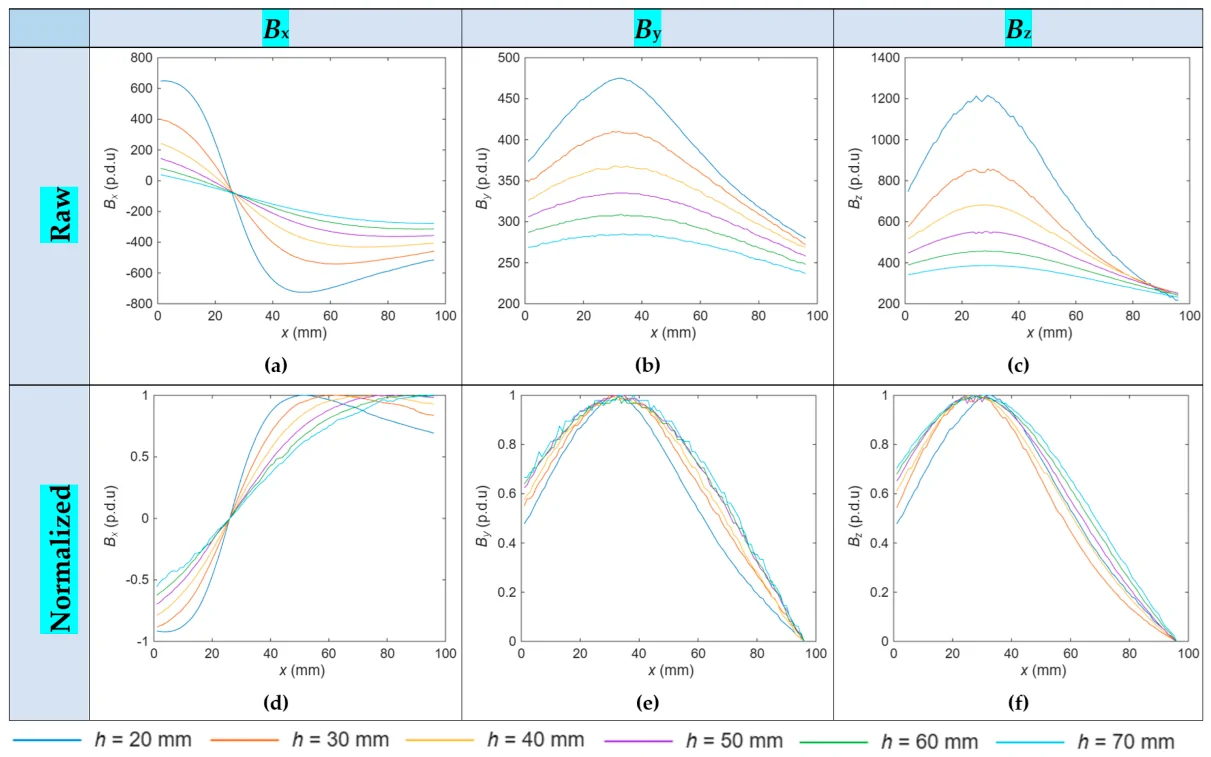
Impact of concrete cover thickness h on waveform: (**a**) RAW data *B*_x_; (**b**) RAW data *B*_y_; (**c**) RAW data *B*_z_; (**d**) normalized data *B*_x_; (**e**) normalized data *B*_y_; (**f**) normalized data *B*_z_.

**Figure 12:**
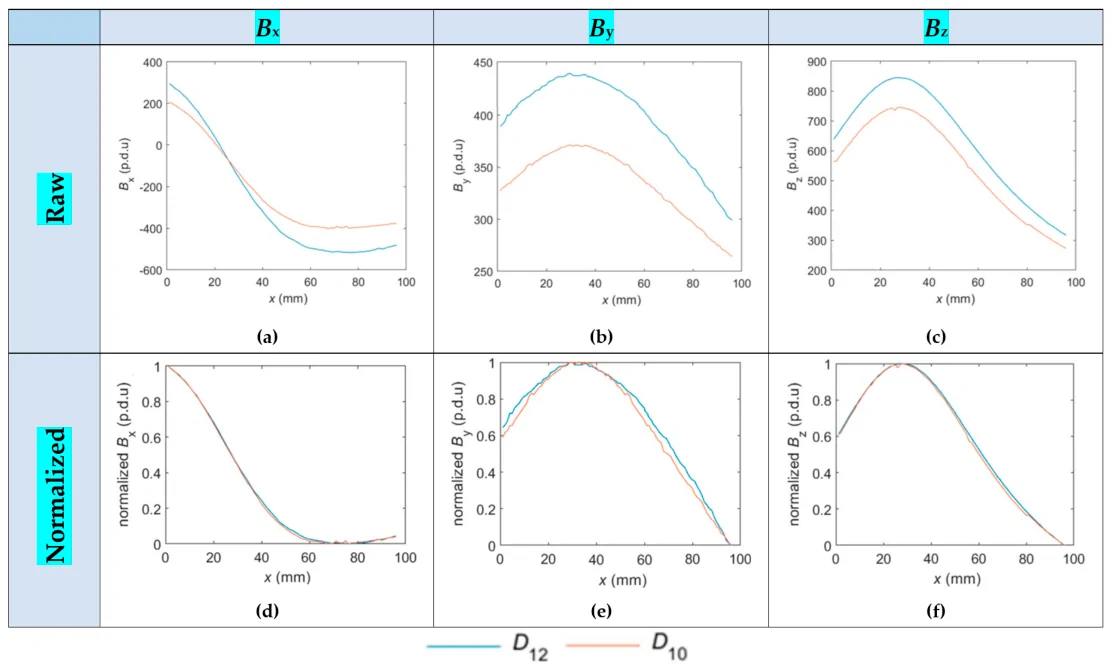
Impact of rebar diameter on waveform: (**a**) RAW data *B*_x_; (**b**) RAW data *B*_y_; (**c**) RAW data *B*_z_; (**d**) normalized data *B*_x_; (**e**) normalized data *B*_y_; (**f**) normalized data *B*_z_.

**Figure 13:**
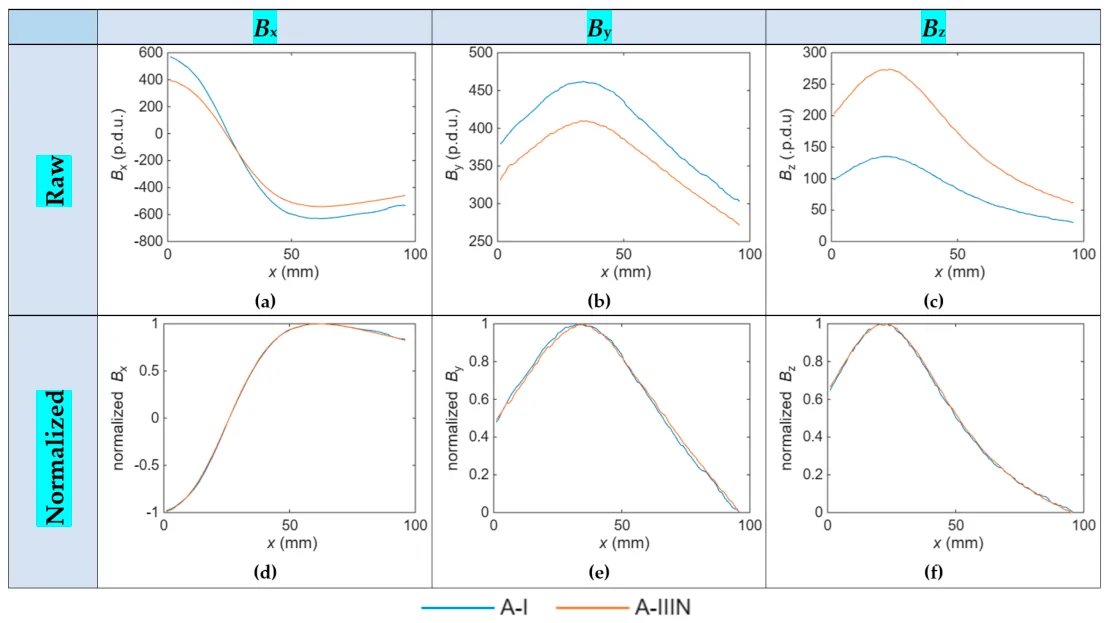
Impact of rebar steel class on waveform: (**a**) RAW data *B*_x_; (**b**) RAW data *B*_y_; (**c**) RAW data *B*_z_; (**d**) normalized data *B*_x_; (**e**) normalized data *B*_y_; (**f**) normalized data *B*_z_.

This section shows raw and normalized MFL waveforms by structural parameter: cover thickness *h* (Figure 11), rebar diameter *D* (Figure 12), and steel class (Figure 13). Normalization, based on ACO decomposition [17], separates amplitude and offset from shape, simplifying interpretation under sparse data.

Figure 11 shows how *h* influences the three magnetic-induction components.

Figure 11 shows that, as thickness increases, most waveform parameters change significantly (especially at low *h*), similar to increased lift-off, which weakens signals and smooths the field. The *B_x_* offset remains constant, aiding decoupling. Minor asymmetries arise from measurement-system inconsistencies.

Figure 12 illustrates how diameter influences every waveform characteristic, especially in *B_y_*, where shape and offset vary. These changes are subtle and noise-sensitive, making diameter hard to distinguish from cover thickness or steel class.

Figure 13 shows how steel class affects waveforms, primarily altering amplitude and offset, while shape remains visually consistent. Subtle shape variations, such as a larger *D* (Figure 12), could be mistaken for small changes in cover thickness and steel class.

As rebars are standardized by mechanical properties, not magnetic ones, bars of the same class from different mills may differ in chemical composition, despite meeting the same grade [78,79], and this compositional variability can alter steel’s magnetic response [80].

Since visual inspection alone cannot reliably decouple the parameters, systematic association-rule analysis is required to identify which signal attributes respond primarily to which parameter, and which remain invariant across the tested configurations.

### 4.2. Signal-to-Noise Ratio

Table 8 shows the median signal amplitude compared to noise amplitude, along with its standard deviation (SD). The results clearly indicate that this ratio generally decreases as the distance between the sensor and rebar increases.

**Figure 14:**
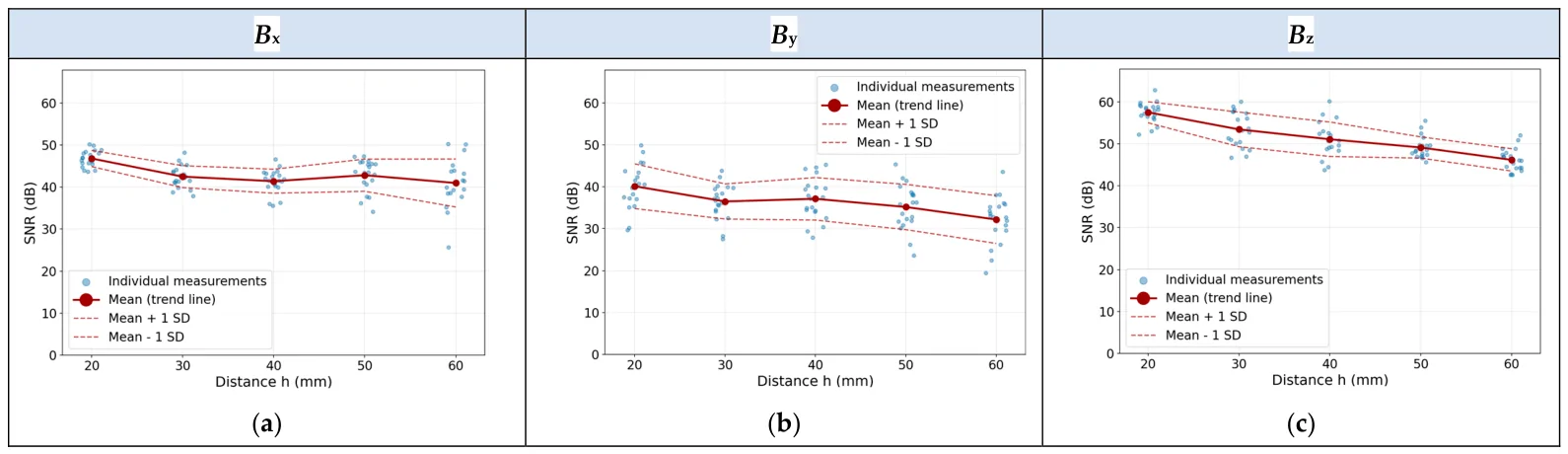
SNR for five cover-thickness levels (20, 30, 40, 50, and 60 mm in 10 mm steps), based on 20 repeated scans: (**a**) *B*_x_; (**b**) *B*_y_; (**c**) *B*_z_.

Figure 14 reports detailed data, showing the signal-to-noise ratio (SNR), calculated by Equation (6), for each spatial component, relative to the distance from the source (rebar) to the sensor. The study combines cover thickness and sensor lift-off into one variable, since concrete’s magnetic permeability is similar to air’s (*μ*_r_ ≈ 1.000002 vs. 1 [81,82]).

SNR decreases overall with increasing distance across all three spatial components, matching expected attenuation from growing *h*. The decline is not perfectly monotonic, but the overall downward trend persists. *B*_y_ has the lowest SNR at all distances; *B*_z_ declines the most in absolute terms, yet remains highest overall; *B*_x_ falls in between. Measurement variability increases, with *B*_x_’s standard deviation growing from smallest to largest distances, indicating more noise as the signal weakens. This observation explains why *B*_y_ was most sensitive to rebar diameter, where shape and offset changes are subtle. The parameter best for isolating diameter operates near the noise floor at practical *h*, making diameter the hardest to decouple.

To confirm that this downward trend is not a sampling artifact, a Spearman rank correlation between distance and SNR was computed (5 distances × 20 scans, *n* = 100). All components exhibited a statistically significant negative correlation (*B_x_*: *ρ* = −0.39; *B_y_*: *ρ* = −0.42; *B_z_*: *ρ* = −0.76; *p* < 0.001), confirming a genuine systemic decline. The rank-based method was selected to assess overall monotonicity without assuming a linear relationship, providing robustness against the local upticks observed in *B_x_* and *B_y_*.

Within tested rebar diameters and steel classes, no consistent impact on SNR was found; the main factor affecting signal quality is the sensor–rebar distance.

### 4.3. Association Rules Analysis (ARA)

To derive decision rules linking signal ACO attributes to structural parameters (*D*, *h*, steel class), continuous measurements must first be discretized. This requires a *sens.min* threshold for each component to distinguish real attribute changes from measurement noise. Although noise affects amplitude, offset, and waveform shape alike, amplitude variation provides the simplest way to quantify it.

The threshold *sens.min* was calibrated from the worst-case standard deviation SD_max_ per component. Comparing two noisy readings combines their variances as $\sigma \sqrt{2}$. Given *n* = 20 scans per condition, a *t*-distribution correction inflated by $\sqrt{1+1/n}$ accounts for the additional uncertainty in the estimated SD, according to (12):

(12) $$k=t\left(19,1-\alpha /2\right)\cdot \sqrt{1+\frac{1}{n}}\cdot \sqrt{2}$$

which yields *k* ≈ 2.51 (90%), 3.03 (95%), and 4.15 (99%). Adopting *k* = 4 provides a round, conservative multiplier at ≈98.8% confidence. This choice is a conservative heuristic that avoids discarding genuine changes at the cost of occasional noise outliers. A full threshold optimization across all parameter combinations is beyond the scope of this methodological study; the selected value is sufficient to demonstrate the decoupling principle, and yields stable rules. Future work will use adaptive selection.

Applying sens.min = 4 × SD_max_ gives component-specific limits (Table 9): *B_x_* and *B_y_* share a similar amplitude scale, while *B_z_* (despite having the lowest relative noise of the three) requires a substantially higher threshold, due to its larger absolute amplitude. A shared threshold was therefore avoided, as it would under-protect *B_z_* or discard valid variation in *B_x_* and *B_y_*.

Assuming normally distributed noise, sens.min offers a solid threshold for attribute discretization, rule induction, and rough-set boundary analysis, but this holds only for the discretized attributes themselves. The underlying ratio presented in Table 8 stays below 0.9% for *B_x_* and 0.5% for *B_z_*, which is negligible next to thresholds an order of magnitude larger. *B_y_* is the exception, reaching 2.3% at *h* = 60 mm, consistent with its role as the weakest, most noise-affected component.

This margin narrows sharply, however, when signals are used to locate the flux-leakage peak, rather than to classify a discretized attribute—a limitation examined in Section 4.4, in the context of diameter identification.

Table 10 presents the association rules obtained through this discretization.

The class effect on O(*B*_x_) is distinguishable from rebar diameter because O(*B*_x_) is invariant with respect to *D* (Table 10, no change at 86% confidence) and with respect to *h* (Table 10, no change at 100% confidence) within the tested configurations, while the same monotonic trend A-I < A-III < A-IIIN was observed across specimens with different diameters.

Rules common across multiple cases tend to be more significant, with their importance scaled by confidence level. For feature decoupling, however, only rules pertaining exclusively to a single parameter are relevant. As Table 10 shows, only steel class affects the offset *O*(*B_x_*) of *B_x_*, making it the sole basis for identifying this parameter.

### 4.4. Multilayer Sensor Decoupled Identification of RC Parameters

Section 4.3 showed that steel class can be isolated using only the ACO attribute O(*B*_x_), demonstrating ARA’s ability to detect exclusive patterns. No such rule exists for diameter *D* or cover thickness *h* among the discretized ACO attributes; these are, instead, identified from peak characteristics outside that set, located as described in Section 3.5. This section examines two characteristics: inter-layer peak position and inter-layer amplitude ratios.

#### 4.4.1. Rebar-Diameter Identification from Inter-Layer Peak Displacement

Locating this peak precisely is not equally reliable at every cover thickness. Near an extremum, the amplitude profile is flat, so point-to-point differences are often below the noise level (Section 4.3), causing the detected peak to shift to a neighboring, noise-elevated point. At small cover thickness, the leakage profile is sharp, so few scan points fall within this flat region, making misidentification unlikely and, when it occurs, limited to one or two measurement points (1 mm spacing). At larger thicknesses *h*, the profile broadens with source-to-sensor distance, placing more points in a noise-comparable range and, in extreme cases, leading to peak-position shifts greater than 5 mm.

**Figure 15:**
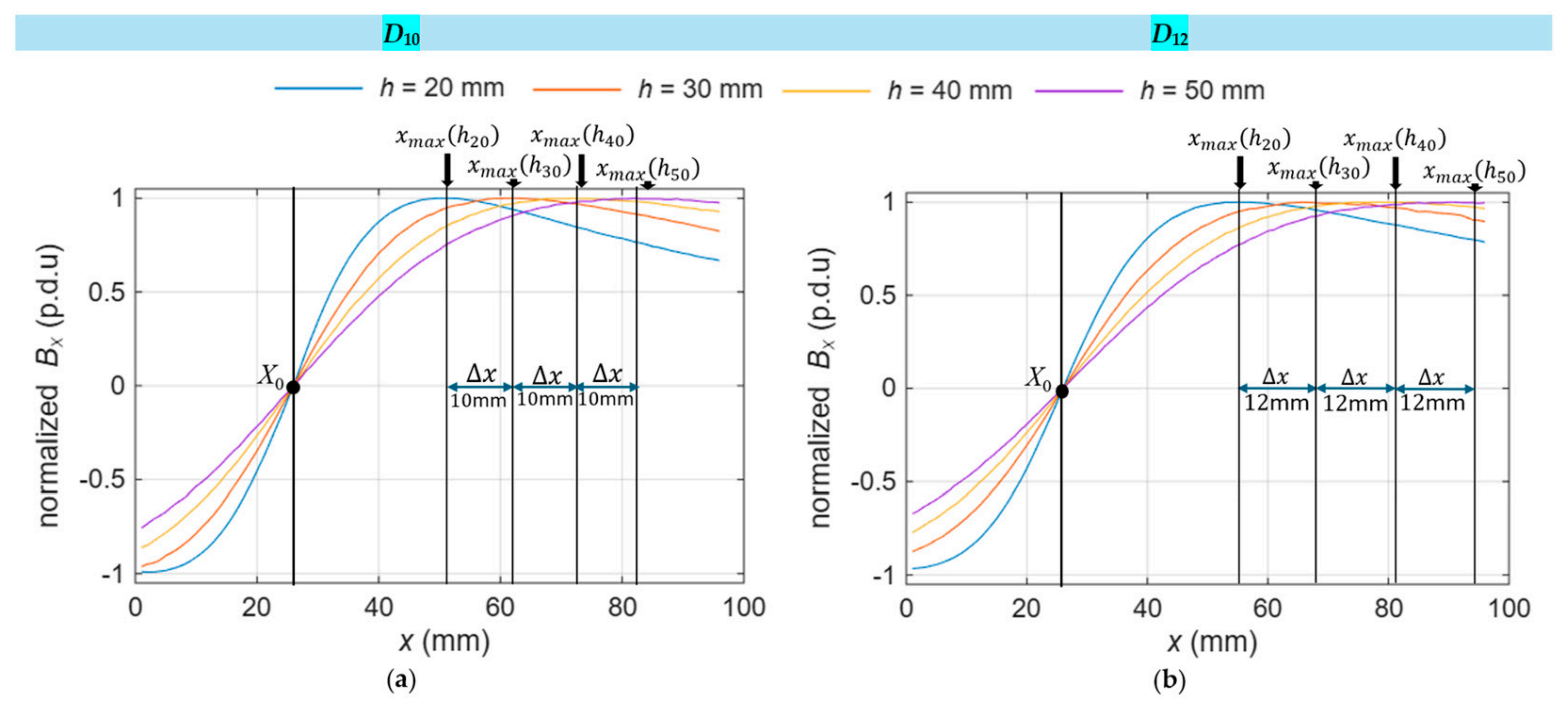
Two-dimensional identification of rebar diameter: (**a**) *D*_10_; (**b**) *D*_12_.

Diameter identification exploits the peak’s lateral position. For a fixed diameter, the *B_x_* waveform along the scan direction peaks at *x*_max_(*h*), which depends on *h*. However, all such waveforms intersect at a common point *x*_0_, providing a fixed reference, independent of *h* (Figure 15). Measuring each peak relative to *x*_0_ across consecutive cover thicknesses defines the inter-layer peak displacement (13).

Δ*x* = *x*_max_(*h_i_*_+1_) − *x*_max_(*h_i_*) (13)

Experiments show that Δ*x* depends only on rebar diameter, remaining approximately constant at *D* = 10 mm for *D*_10_ and 12 mm for *D*_12_, regardless of *h* (Figure 15). The spacing between the transducer’s three sensor layers (*n* = 5 mm, as in Figure 5) was chosen specifically, so that this same relationship holds for the layers’ simultaneous readings: the resulting inter-layer displacements, Δ*x*_12_ (layers 1–2) and Δ*x*_23_ (layers 2–3), each provide an independent estimate of *D*, and are averaged for robustness. Because Δx is, by construction, geometric rather than amplitude-based, it showed no measurable dependence on *h* across the tested range, yielding a diameter estimate invariant with respect to concrete cover thickness across the tested range. The subsequent analysis presents a detailed statistical evaluation of the diameter estimates, including measurement repeatability and SNR effects.

The positional uncertainty described above propagates directly into this estimate, since *D* is derived from the separation between two independently localized peaks, rather than a single one; the Discussion and Evaluation Sections discuss its practical impact on identification accuracy at larger cover thicknesses.

#### 4.4.2. Cover-Thickness Identification from Multilayer Peak Amplitude

**Figure 16:**
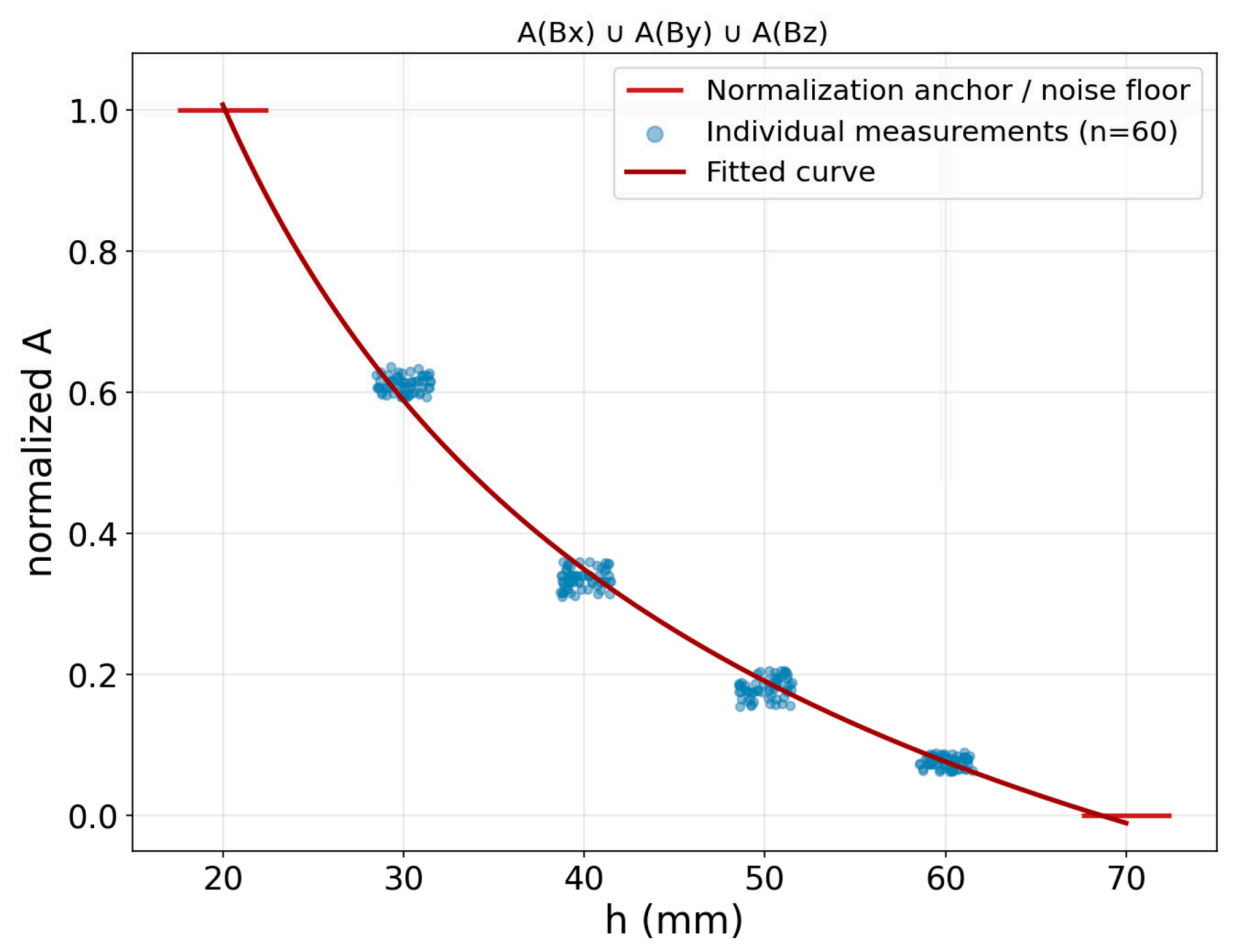
Normalized relationship between cover thickness and signal amplitude.

Amplitude is crucial for signal identification, but is often affected by many factors (Table 10), especially outside laboratories. Normalized amplitude versus sensor-to-source distance, however, tends to follow a consistent shape. Because each component (*B_x_*, *B_y_*, *B_z_*) is normalized to its own min–max, the shared endpoints (1 at *h* = 20 mm, 0 at *h* = 70 mm) follow by construction; real evidence lies in the close, low-scatter agreement of the three curves at the interior, measured points (30–60 mm, Figure 16), invariant with respect to *D* and steel class within the tested range, as demonstrated by ARA, and further stabilized by pooling all three spatial components, rather than relying on a single one. This shared curve is described by the following empirical approximation (14):

*A*(*h*) = 12.03 ⋅ *h*^−0.6181^ − 0.8814 (14)

Equation (14) is an empirical fit to measured data over the typical tested range of *h* (20 to 60 mm), not a physically derived model. The negative intercept is a fit artifact and does not affect thickness estimation within this range. To assess generalization beyond the fitted data, Equation (14) was additionally evaluated by leave-one-configuration-out cross-validation [83,84]: for each of the 20 specimen configurations in turn, all its measurements were withheld, the model was refitted on the remaining 19 configurations, and the withheld measurements were predicted from the refitted curve. Out-of-sample errors are reported alongside in-sample errors in Section 4.5.2.

Although the relationship is monotonic, its strong nonlinearity means that a single absolute-amplitude measurement cannot uniquely determine the corresponding position on the curve without a reference. This reference is provided by the transducer’s three vertically stacked sensing layers, spaced *n* = 5 mm apart. The simultaneous absolute-amplitude readings *A*_1_, *A*_2_, and *A*_3_ are calculated as follows (15):

(15) $$\left\{\begin{array}{l} A_{1}=12.03\cdot h^{-0.6181}-0.8814\\ A_{2}=12.03\cdot {\left(h+n\right)}^{-0.6181}-0.8814\\ A_{3}=12.03\cdot {\left(h+2n\right)}^{-0.6181}-0.8814 \end{array}\right.$$

Because the closest layer generally produces the highest amplitude under the assumed monotonic decay, the readings are normalized, with respect to *A*_1_. The normalized amplitudes are therefore defined as $a_{1}=\frac{A_{1}}{A_{1}}=1,\;a_{2}=\frac{A_{2}}{A_{1}},\;a_{3}=\frac{A_{3}}{A_{1}}.$

These two amplitude ratios contain the information used to estimate the cover thickness. Since the identification relies on relative attenuation, rather than on the absolute amplitude scale, the method may be less sensitive to variations in alloy composition or measurement gain.

Cover thickness *h* is estimated by minimizing the sum of squared residuals between measured and model-predicted ratios via a 1D non-linear least-squares search, avoiding direct algebraic inversion. While a single ratio (two layers) suffices for a linear system, the pronounced non-linearity of Equation (15) makes single-ratio inversion highly noise-sensitive, especially in flatter-curve regions. The third layer adds an independent constraint, identifying the value of *h* that satisfies both ratios simultaneously. Figure 16 illustrates this normalized amplitude–distance relationship. The boundaries at *h* = 20 mm and *h* = 70 mm represent the normalization anchor (closest distance, where amplitude is 1) and the practical noise floor, respectively, rather than empirical distributions.

To quantify universality, we computed the root-mean-square deviation (RMSD) between measured normalized amplitudes and the fitted curve for each diameter–class combination. The RMSD remained below 0.05 in all cases, confirming that the normalized response is invariant, with respect to *D* and steel class, across all tested configurations.

### 4.5. Decoupled Evaluation of RC Parameters

The three structural parameters are decoupled and identified using methods suited to their categorical or continuous nature, and are covered individually in the following subsections.

#### 4.5.1. Evaluation of Rebar Class

Unlike the association rule, which uses pairs of records differing by one parameter, class discretization here uses the base database directly (each of the 400 measurements paired only with its offset O(*B*_x_) and class label).

The measured offset values span approximately 74–97 p.d.u. across the three classes, consistent with the rule IF class↑ THEN O(*B*_x_)↑ relationship established in Table 10. Before any discretization, the overlap coefficient between adjacent-class O(*B*_x_) distributions confirms strong separation (OVL ≈ 0.02 for A-I–A-III, ≈ 0.03 for A-III–A-IIIN).

This range was divided into ten equal-width bins (step ≈ 2.3 p.d.u.), and each measurement was assigned to its nearest bin (bin assignment is presented in Table 11). Because the classes are well separated, this fixed-step discretization effectively reduces to a three-class split, with A-I occupying the lowest bin, A-IIIIN the highest, and A-III falling in between.

Misclassified records mainly occurred at small cover thicknesses (*h* = 20 mm), where the measurement system’s asymmetry more strongly influences the crossover point. This reflects a geometric bias, not noise, and repeated measurements with different alignments can correct it. No filtering can fix this. As part of the protocol, asymmetric results for specimens with small concrete cover were checked by verifying alignment and remeasuring, when needed. Following this procedure, all records matched their correct class labels: *α*_B_(*X*) = 1.0 for all classes, and *γ*_B_(P) = 1.0. The number of corrected measurements is presented in Table 11.

Reprocessing O(*B*_x_) with median filtering and GA approximation does not alter the outcome, as expected: the crossover point is a fixed reference, not a peak, and raw exceptions result from geometric bias, not noise, so filtering does not address the root cause.

Taken together, OVL shows the underlying offset distributions are well separated at the population level, while rough-set accuracy shows that, once discretized, measurements are assigned deterministically.

Magnetic signatures may vary across mills for the same mechanical grade (Section 4.1). Nevertheless, the strong separation observed in the present dataset (OVL ≈ 0.02–0.03, *α*_B_(*X*) = 1.0) indicates promising potential for steel-class estimation under the tested laboratory conditions, despite the compositional variability represented in the experiment. Unlike cover thickness, which relies on normalized waveform shape, and diameter, which relies on inter-layer peak position (Section 4.4.1), class identification depends primarily on an absolute signal value. Because compositional variability may affect signal magnitude more directly than waveform shape or spatial position, steel-class identification lacks the same invariance argument as the other two parameters.

#### 4.5.2. Evaluation of Concrete Cover Thickness

In contrast to steel-class identification, which depends on a fixed signal value, cover-thickness estimation leverages the normalized waveform shape—linking the estimate to a specific physical phenomenon, rather than an absolute reading, in keeping with this work’s XAI orientation.

**Figure 17:**
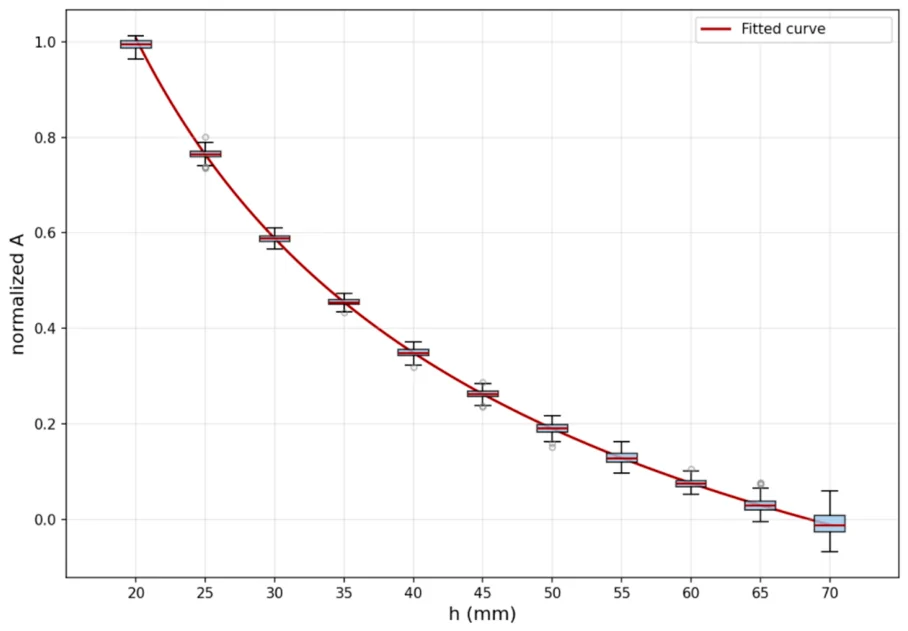
Distribution of normalized amplitude by cover thickness across the 5 mm grid (*n* = 240 per level); box edges show interquartile range, whiskers full range excluding outliers (circles). The solid line is the fitted one (14).

Each combination of spatial component, diameter, and steel class was normalized separately, anchored to the mean amplitude of the two extreme cover-thickness classes (20, 60 mm), rather than a single raw reading. This gives *n* = 240 at each of the five tested thicknesses (Table 7). The three-layer sensor extends this to a 5 mm grid (20–70 mm) via simultaneous three-lift-off readings; these virtual points share their originating scans, and are therefore not fully separate observations. The analysis pools all three spatial components—a worst-case choice, since *B*_y_ has the weakest SNR (Section 4.2) and *B*_x_ alone would likely perform better; if the method holds under the least favorable component, its practical reliability is better supported. Results were pooled for the analysis below only after this per-subgroup normalization (Figure 17).

At the coarse, physically tested resolution (10 mm), adjacent classes do not overlap (OVL < 0.001 for all pairs), so the rough-set formalism is trivial and is not pursued further at this resolution. Table 12 shows the finer 5 mm grid enabled by the layered sensor.

Overlap stays negligible up to 60 mm (finer than the typical 10 mm resolution), rising sharply only at the outer edge, where several sources of variability compound near the sensor’s range limit. This tight clustering across 30–60 mm also supports the interior-point agreement underlying the shared curve of Section 4.4.2.

Beyond this resolution, *h* is better treated as continuous. Because the curve (14) is strongly non-linear, normalized-amplitude spread alone is not informative about real-world accuracy: the same spread implies far greater uncertainty in h where the curve is flat than where it is steep. Millimeter-level accuracy was therefore obtained by solving the three simultaneous layer readings for h via least squares (15) and comparing the result to the known true *h*. Table 13 reports the resulting error by cover thickness.

Figure 17 shows normalized-amplitude distributions, with expanding boxes at both ends indicating increasing inversion errors, consistent with Table 13. The model curve was extrapolated from 60 to 70 mm because it remained consistent with the observed trend; this range is not experimentally validated. Accuracy is best at *h* = 20 mm (in-sample RMSE = 0.16 mm, out-of-sample 0.24 mm), degrades gradually through the mid-range, and worsens sharply beyond 50 mm. The ratio between out-of-sample and in-sample errors grows with h, from approximately 1.5× at 20 mm to 2× at 60 mm, consistent with the SNR degradation documented in Section 4.2 and the error propagation in Equation (16). Beyond roughly 60 mm, the inverted estimate mainly suggests that the cover thickness is near the sensor’s practical limit, rather than providing an accurate measurement. The absolute estimation error correlates with cover thickness (Spearman *ρ* = 0.603, *p* < 0.0001, *n* = 1200 triangulated estimates over the five physically tested levels; virtual 5 mm grid points excluded).

This trend’s mechanism is the same non-linearity as that noted above: propagating amplitude noise through the model’s derivative via the delta method, given as (16):

(16) $$\sigma_{h}\approx \frac{\sigma_{A}}{|\mathrm{dA}/\mathrm{dh}|}$$

Equation (16) shows that as |d*A*/d*h*| flattens with *h* while *σ*_A_ stays roughly constant, the same absolute noise yields disproportionately larger uncertainty in *h*—without any increase in raw-measurement noise.

Only the positive half of the amplitude–distance relationship is used here; fitting the negative half independently, rather than jointly, could further improve accuracy, and is left for future work.

#### 4.5.3. Evaluation of Rebar Diameter

Rebar diameter is determined from Δ*x*, the inter-layer peak displacement (Section 4.4.1). This position-based feature is robust to variations in alloy composition and is calculated from *B*_x_, which provides a strong SNR (Table 8). Overlap arises from the genuinely small physical difference in peak position between adjacent diameters. The rebars *D*_10_ and *D*_12_ are not only among the most common, but also adjacent in the standard size series, making them the most suitable and conservative test for discrimination (larger gaps ease separation).

Unlike steel class and cover thickness, which barely overlap, diameter exhibits significant overlap, even after processing—demonstrating the usefulness of the full rough-set formalism, and serving as an illustrative case of the proposed methodology.

**Figure 18:**
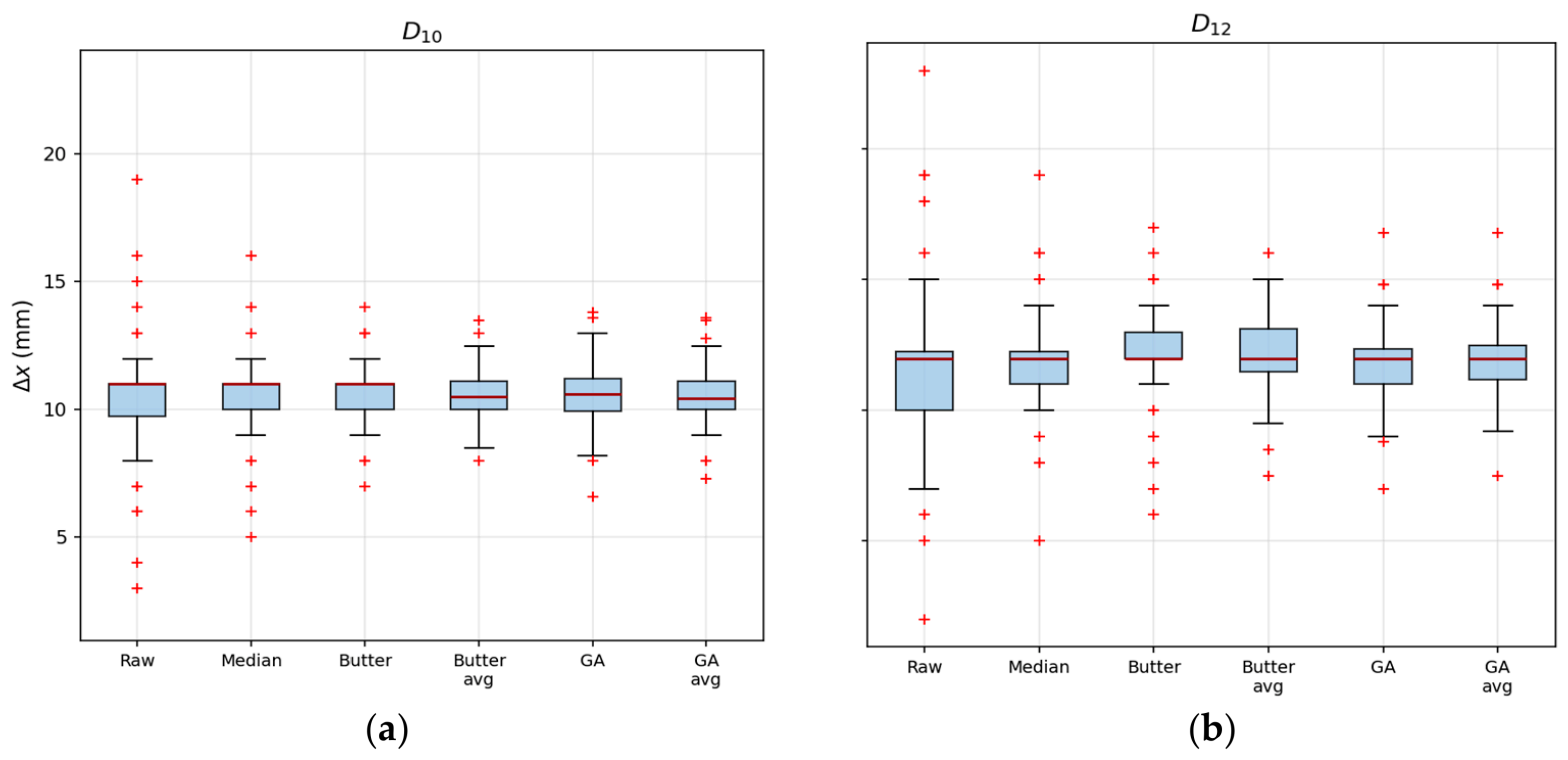
Δ*x*(*D*_10_) and Δ*x*(*D*_12_) across six processing stages (raw, median-filtered, Butterworth-filtered, Butterworth-filtered averaged, GA-fitted, GA-fitted averaged): (**a**) *D*_10_; (**b**) *D*_12_.

The values of Δ*x*(*D*_10_) and Δ*x*(*D*_12_) were determined at four processing stages: raw, median-filtered, GA-fitted, and GA-fitted with averaging (Δ*x*_12_, Δ*x*_23_). These were compared to Butterworth-filtered data, including versions with inter-layer averaging. Over these stages, the distributions become narrower, while the medians stay close to the true diameters; however, some large outliers still occur, even after processing (Figure 18).

Analysis of Δ*x* shows that OVL typically decreases across processing stages, but not linearly. The GA-fitting stage briefly exceeds the Butterworth-averaged value before the averaging step in the GA process, leading to the lowest overall overlap. The Mann–Whitney test indicates significant differences between distributions at each stage, including the raw data, where overlap remains substantial. This demonstrates that population-level significance alone does not guarantee reliable practical classification, as highly overlapping distributions can still differ significantly (Table 14). Therefore, a per-measurement metric is necessary.

Rough-set quantities were calculated by binning Δ*x*, according to the resolution, at each processing step. The initial resolution was 1 mm (RAW, median, and Butterworth filters), reduced to 0.5 mm after inter-layer averaging of Butterworth-filtered data; GA-fitting combined with subsequent averaging yielded 0.1 mm, a tenfold increase over the raw baseline. As shown in Table 15, this resolution enhancement is more significant for the rule-based “glass-box” identification scheme than noise reduction alone, as it allows the rough-set approximations to better differentiate parameter-driven signals from measurement uncertainty at a finer scale.

At 1 mm resolution, no Butterworth-filtered *D*_10_ measurement falls entirely within a *D*_10_ bin (*α*_B_(*D*_10_) = 0), indicating that the two classes are nearly co-located. Simple inter-layer averaging increases *γ*_B_(*P*) from 0.067 to 0.125. The largest improvement occurs with GA-fitting at 0.2 and 0.1 mm resolution, suggesting that its advantage stems from improved discretization for classification, rather than noise reduction, leading to better diameter accuracy. Table 15 shows that reducing bin width from 1 mm to 0.1 mm increases *γ*_B_(*P*) from 0.067 to 0.600, while Butterworth at 1 mm yields only 0.058 to 0.067. Finer discretization, not noise suppression, improves classification.

**Figure 19:**
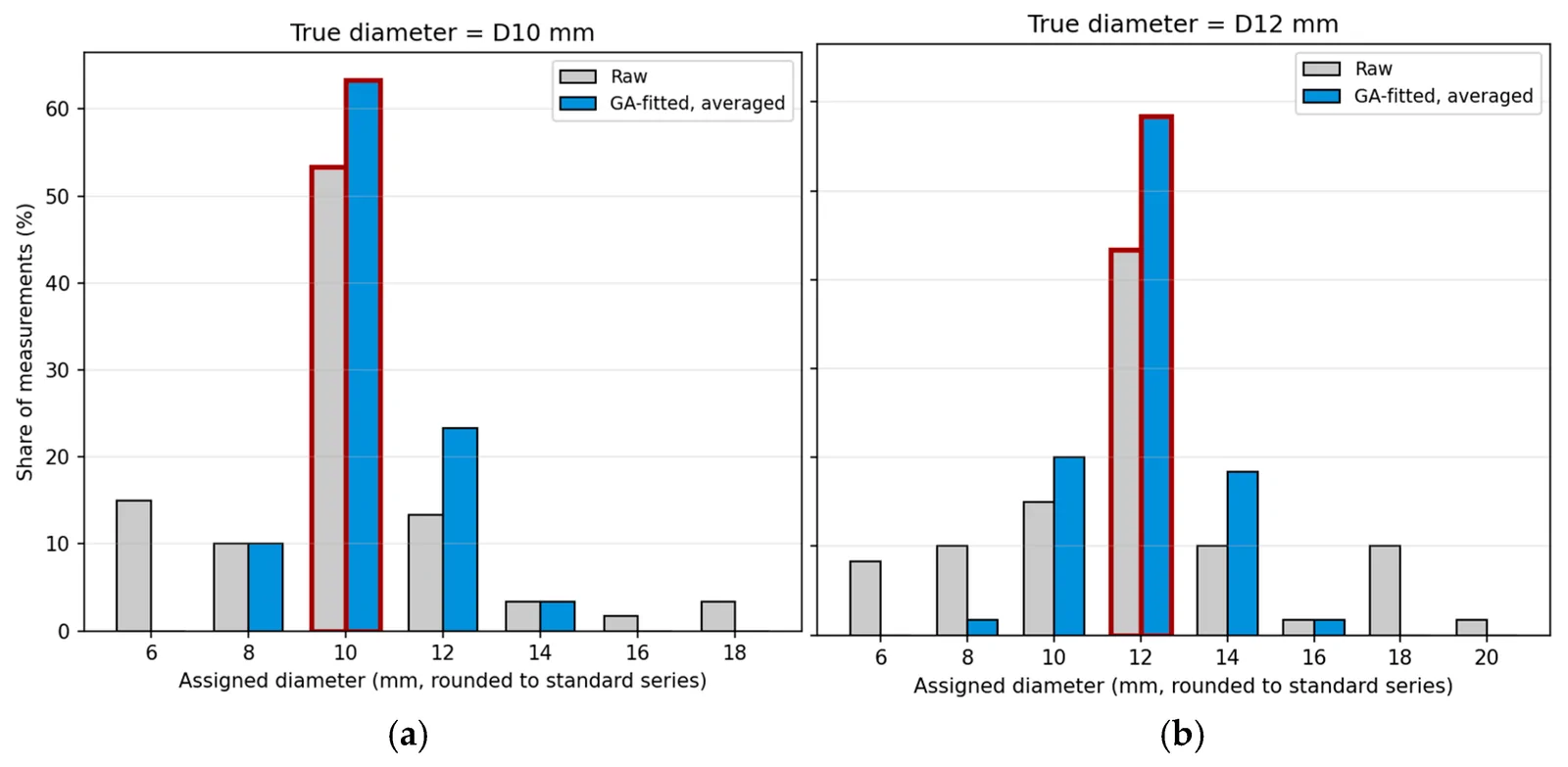
Share of measurements assigned to each standard rebar diameter after rounding, for true *D*_10_ and *D*_12_, comparing raw and fully processed (GA-fitted, averaged) data: (**a**) *D*_10_; (**b**) *D*_12_.

Rounding each measurement to the nearest standard rebar diameter provides a more standard view of classification accuracy (Figure 19).

As a methodological study, transparency, simplicity, and parameter decoupling were prioritized over maximizing accuracy; the results demonstrate the pipeline, rather than peak performance. Standard diameter classification yields 63.3% for *D*_10_ and 58.3% for *D*_12_ (averaging ~60% across 20–60 mm), with the 40–60 mm range proving particularly challenging (and often omitted in the literature), due to low SNR. After processing, RMSE drops to 1.37 mm (*D*_10_) and 1.44 mm (*D*_12_), down from 2.57 mm and 3.25 mm, on raw data. Processing effectively eliminates gross errors (which reached from 6 mm to 18–20 mm in raw data), limiting remaining misclassifications almost entirely to adjacent sizes (*D*_10_ vs. *D*_12_).

Two distinct metrics converge at the final stage: the conservative zero-overlap bound *γ_B_*(*P*) = 60% and the nearest-diameter accuracy (≈60%). Though fundamentally different (*γ_B_*(*P*) measures instances in pure decision bins, while accuracy counts correct sides of the decision boundary), their agreement stems from the same physical constraint: overlapping Δ*x* distributions between *D*_10_ and *D*_12_. Because 0.1 mm discretization and standard-size rounding establish nearly identical boundaries, the two values coincide. This reveals the fact that roughly 40% of measurements are ambiguous, meaning higher accuracy requires reducing distribution overlap, not tuning classification rules.

In engineering practice, avoiding major errors is often more critical than exact classification. Accepting assignments to the exact or an adjacent size raises accuracy to ~97% for both *D*_10_ and *D*_12_ (up from 76% and 68% on raw data). The correct-or-adjacent figure is a gross-error rate rather than an accuracy: it reports how often the method avoids an error larger than one step in the standard-diameter series. An error of one standard size changes a load-capacity estimate modestly, whereas an error of two or more sizes changes it materially. Diameter-identification accuracy is the exact-class figure of approximately 60%, reported above. This dramatic reduction in gross misclassifications provides strong practical utility. While adding signal descriptors or complex algorithms could improve results, they were omitted in order to keep the workflow simple and low-complexity.

## 5. Discussion

This section interprets the results presented in Section 4, relates them to the research questions (Table 3), and positions them within the broader context of the literature reviewed in Section 2. The discussion addresses the methodological implications of the results, key limitations arising from sparse data and transparency priorities, and promising directions for future work.

### 5.1. Interpretation of Results

The combined techniques may be applicable beyond reinforced-concrete identification. to other small-data NDT problems. ACO decomposition can also serve as a pattern-recognition tool for other small-data NDT problems, as it compares new measurements to a single reference sample and analyzes deviations without a comprehensive rule database [17,23,41]. When ACO alone cannot separate a parameter, association rule analysis (ARA) can be applied to other waveform features (Section 4.4.1 and Section 4.4.2). ARA is especially effective for sparse databases, as it finds all rule-supported data relationships within data pairs without needing a large sample set. Rough-set theory and the overlap coefficient complement this rule-based method (Section 4.5), providing population-level and individual measurement confidence bounds, without assuming specific data distributions. Curve-fitting techniques such as GA-based approximation provide an additional benefit, by increasing the effective measurement resolution (Section 4.5.2 and Section 4.5.3) without new data collection, especially when measurement, not analysis, is the main bottleneck.

Global search trends (Introduction, Figure S11) show a sharp rise in interest in small-data methods, while academic output in the two established fields has plateaued (Figures S12 and S13). This gap suggests that demand is driven largely by practitioners (engineers and developers), rather than by the research community alone. Accordingly, the pipeline relies on human-readable rules that require only a few reference measurements for calibration, enabling field engineers to refine the workflow incrementally, without machine-learning specialists or years of data collection.

The growth of methods such as multitask learning, decoupling, and rule-based inference (Table S2) reflects a recognition that sparse-data settings need a different design philosophy. Yet the underlying constraint remains: classical black-box methods, including multitask learning, must estimate shared representations from limited task data, with sample needs that increase with task count and representation dimensionality. This is the curse of dimensionality in practice: more parameters multiply the data burden, without adding information. Feature-level decoupling, instead, avoids it structurally, reducing a three-parameter problem to three single-parameter tasks, each identified by a physically interpretable attribute, rather than a shared learned representation.

This work prioritizes data efficiency and transparency over black-box performance. Compared with [37] (RMSE 0.91 mm for diameter, 0.87 mm for cover), our pipeline achieves comparable cover accuracy at low-to-moderate *h* (out-of-sample RMSE 0.24–0.97 mm up to *h* = 50 mm) but degrades at *h* = 60 mm (2.02 mm), and lower diameter accuracy (1.37–1.44 mm after full processing). Diameter identification may require the refinements in Section 5.3. For concrete cover thickness, Section 4.5.2 and Section 4.5.3 suggest that interpretability needs come with a cost in accuracy, though a direct comparison remains as future work. The moderate exact-diameter accuracy reflects limited feature resolution (measurement noise, sensor-to-rebar distance, and small differences among neighboring diameters), rather than cross-parameter interference. Accepting the correct or adjacent standard-diameter class raises accuracy to approximately 97%.

Every path to higher accuracy has costs, deliberately left unexploited for simplicity and transparency: modeling the waveform’s lower half would lengthen measurement time; adding sensor layers to improve diameter estimation increases transducer complexity and, because they are progressively farther from the rebar, introduces extra noise at large *h*; finer GA sampling sharpens discretization at the cost of longer measurement or processing; and a higher-order curve model (14) could improve accuracy, especially for diameter, but was avoided for simplicity. These trade-offs (accuracy versus hardware, or interpretability) are intentional, with reconsideration possible when accuracy is paramount. In these terms, the choice is a quality-management decision, rather than a purely algorithmic one: the pipeline sets the accuracy level the intended inspection task needs, and spends the remaining budget on traceability, rather than maximizing accuracy at the cost of evidence an auditor can follow.

The results support the qualitative comparison in Table 4 and Figure 3, and address RQ4: deterministic steel-class classification (*α*_B_(*X*) = 1.0) provides “transparent by design” interpretability, unlike the post hoc explanations required by MTL. Independent, parameter-specific metrics for all three parameters (Section 4.5.1, Section 4.5.2 and Section 4.5.3) show that errors are confined to a single parameter, rather than spreading through a shared latent space [53]. These results were obtained with 400 measurements across 20 configurations (below the scale at which MTL is reported to work effectively [52]). This aligns with feature-level decoupling: the method is calibrated using a few reference configurations, not trained on a labeled dataset, which is its intended mode of operation. Whether it requires less data than MTL under matched conditions, remains to be tested in a controlled comparison.

### 5.2. Limitations

The limitations mainly highlight this work’s focus on sparse data and transparency, not oversights:

1. The tested range (*D* = 10–12 mm, *h* = 20–60 mm) is narrow, reflecting the NDT reality that exhaustive coverage is rarely feasible under data scarcity; identifying interpolable rules (TRL 4) is the practical alternative, favoring simple, field-refinable patterns over large-database paradigms (a deliberate trade-off). Generalization beyond this range requires further validation.
2. Within these ranges, the reported relationships are interpolations bounded by measurement at the extreme levels; the Section 4.5 accuracy figures apply only to this interval. Interpolation relies on physical mechanisms linking each feature to its target parameter, not on measurement-grid density. Beyond these ranges, they are extrapolations supported by physical reasoning, rather than by experiment: the amplitude–cover relationship should remain monotonic above 60 mm, but the signal-to-noise degradation documented in Section 4.2 will dominate before it ceases to hold, and the inter-layer displacement relationship should extend to larger diameters only while the bar diameter remains small, relative to the 5 mm inter-layer spacing.
3. No dedicated simulation was conducted for the entire pipeline (even though linking waveform features to physical phenomena is important for sparse data); it focused only on the fundamental physical relationships [17,23,41]. Beyond that, the method relies on basic parameters such as peak amplitude, offset, and correlation, which do not require further validation.
4. The sensitivity of *k* = 4 in *sens.min* to other values was not tested. However, it defines a deliberately safe zone (≈98.8% confidence) minimizing confusion with the neighboring class, consistent with minimizing risk under sparse data.
5. GA hyperparameters were set experimentally, not tuned. Transparency comes from the closed-form model (14), not the fitting process, so tuning does not affect interpretability. Extensive tuning with sparse data risks fitting noise.
6. Equation (14) was fitted on all available data; its in-sample residuals in Table 13 are therefore optimistically biased. Leave-one-configuration-out cross-validation gives out-of-sample RMSE values approximately 1.5–2× larger, rising from 0.24 mm at *h* = 20 mm to 2.02 mm at *h* = 60 mm, with the largest degradation where SNR is lowest. Because repeated scans of one specimen are not independent observations, a random k-fold split would not have removed that bias; withholding an entire configuration, as done here, ensures that no measurement contributes to the model that predicts it. The reported accuracy should therefore be read as an upper bound within the tested range, and the out-of-sample figures as the appropriate estimate for new specimens.

### 5.3. Future Work

Beyond the trade-offs and limitations above, several broader directions remain open:

1. Validate across a wider *D*/*h* range with a sensitivity analysis of *k*, to clarify how far current rules and thresholds generalize.
2. GA-based approximation proved valuable (Section 4.5.2 and Section 4.5.3); a broader study of curve-fitting algorithms and their trade-offs is a natural next step.
3. Extend the leave-one-configuration-out validation to specimens outside the present set, to test whether the out-of-sample accuracy holds for unseen configurations.
4. Model the waveform’s lower half, and add sensor layers for diameter (Section 4.5.2 and Section 4.5.3), trading transparency for accuracy where that balance is preferable.
5. Conduct a dedicated, quantitative comparison against MTL baselines as a separate study.
6. Apply the pipeline to areal, scan-based inspection, including AMR sensor arrays, beyond the point-measurement setting used here.
7. Generalize the rough-set framework (Section 3.7) into a full expert system spanning multiple defect types.
8. Extend ARA to higher-order, multi-parameter rules, fuzzy boundary regions, and active refinement, guided by the boundary region itself.
9. Complement this work’s methodological, data-efficiency focus with a broad, dedicated ablation study across all three parameters.
10. Assess the stability of reducts across random sub-tables, following the dynamic-reduct approach [75], to establish how far the identified rules generalize beyond the present dataset.

## 6. Conclusions

This work addressed four research questions on decoupling structural parameters from sparse MFL measurements:

RQ1—the analysis revealed which features depend primarily on one parameter and are not measurably affected by the others within the tested configurations. Only the steel class is identified through the ACO attribute O(*B*_x_), which is sensitive to variations in alloy composition. Conversely, diameter and cover thickness are derived from geometric features (inter-layer peak position and normalized waveform shape) that showed no measurable dependence on the other parameters within the tested configurations.

RQ2—the question was how to derive association rules from limited reference measurements. The proposed approach integrates components that, together, handle sparse data. Association-rule analysis combined with the statistically grounded sens.min threshold and rough-set confidence bounds (Section 3.6) yielded rules with support and confidence values that characterize the internal consistency of the rules on the 400 measurements from which they were derived (Table 10). Because no independent set of unseen specimens was available, these values should be read as in-sample rule strength, rather than as a generalization estimate. Validation on specimens outside the present set is identified as the primary next step (Section 5.3).

RQ3—this asked how the three tasks can be decoupled without a shared latent space. Each parameter is identified through an independent mechanism and evaluated with its own parameter-specific metric (Section 4.5.1, Section 4.5.2 and Section 4.5.3), avoiding the shared representation (and the negative transfer it can invite) inherent to MTL approaches.

RQ4—the question was how this decoupling compares to MTL, regarding interpretability and noise robustness. This was addressed through a systematic literature review and conceptual comparative analysis (Section 2, Table 4, Figure 3), complemented by empirical evidence characterizing the proposed pipeline, in Section 5.1. The results suggest potential benefits in terms of transparency and data efficiency relative to shared-representation learning, although direct quantitative comparison remains beyond the scope of the present study, and is deferred to future work.

The reported rules were obtained from 400 measurements across 20 configurations without labels, for every task. They support the pipeline as a transparent alternative to shared-representation learning for multi-parameter identification from sparse NDT measurements, demonstrated here on RC parameter identification as a broader example. A quantitative comparison against multitask baselines trained on the same data under matched conditions remains as future work. Beyond the measurement problem, the result carries a quality-assurance implication: because each parameter is read from a named signal attribute through a human-readable rule, an inspection outcome can be documented, challenged, and re-derived by a third party, a property that black-box identification does not provide and that conformity assessment of existing structures requires. This is the practical dimension that the worldwide search-interest trends in the Introduction point to: the demand comes from engineers and developers, and the pipeline is designed to be usable by them.

The growth of method families such as multitask learning and decoupling (Table S2) shows that the sparse-data problem is widely recognized. Classical black-box methods estimate a shared representation from limited task data, requiring more samples as task count and representation dimensionality grow. Feature-level decoupling reduces this burden by splitting the three-parameter problem into three single-parameter tasks, each tied to a physically interpretable signal attribute. Data scarcity is structural, not incidental: the review confirms it (Section 2.2), and the pipeline is designed for it.

## Figures and Tables

**Table 2:** Challenges limiting the adoption of AI-based NDT in reinforced concrete.

Challenge	Description	Consequence for AI-NDT of RC	Refs.
Data scarcity	Labeled RC data are costly; benchmark datasets are limited	Poor generalization across structures	[24,46]
Interpretability	Deep models may be opaque; post hoc explanations differ from by-design interpretability	Limited traceability in safety-critical structural assessment	[16,25,47,48]
Parameter interdependence	Responses to different parameters overlap	Cross-parameter interference; unreliable single-parameter inference	[29,30,37]
Simulation-to- reality gap	Synthetic-trained models degrade on real objects	Reduced field accuracy; motivates physics-informed transfer	[28,36,49]
Lack of standards	No unified collection or evaluation protocol	Limited comparability across studies	[3,4]
Measurement uncertainty	NDT signals carry noise and calibration error	Deterministic mappings may degrade; probabilistic approaches can better accommodate uncertainty	[2,7]

**Table 3:** Research questions, supporting literature, and their contribution.

RQ	Question	Motivating Evidence	Corresponding Contribution
RQ1	Which MFL features (ACO attributes or geometric relationships) are sensitive to one parameter only and invariant to the others?	Coupling in eddy-current and pulsed-eddy-current NDT [29,30,37]; invariance metrics [50,51]	ACO decomposition and multilayer peak-geometry features exposing single-parameter attributes [17,41]
RQ2	How can association rules identify such relationships from the few reference measurements?	Rule mining [40], rule ensembles [39]; unstable importance estimates at small *n* [35]	Association-rule procedure reporting support and confidence [23]
RQ3	How can the three tasks be decoupled without a shared latent space?	Negative transfer in MTL [52,53]; unsupervised disentanglement not achievable without inductive bias [54,55]	Transparent-by-design three-subtask scheme
RQ4	How does feature-level decoupling offer interpretability and signal its uncertainty in the context of measurement noise, and how do these properties compare qualitatively with MTL approaches in the existing literature?	Post hoc explanations may be unstable [25,47]; black-box models lack built-in confidence signals [16]; probabilistic treatment of measurement uncertainty provides a reference point [2,7]	Qualitative comparison with MTL baselines based on literature review; deterministic rules vs. run-to-run variance; uncertainty signaled via BN_B_(X) variation with SNR

**Table 5:** Method × domain co-occurrence in the 235-record corpus: number of records containing both concepts. Empty cells (shown in bold red) indicate combinations with no records in the corpus—precisely the gaps that this work addresses. Columns combine domain, modality, and task tags.

	Reinforced Concrete	Rebar Ø/ Cover	Magnetic Flux Leakage	Eddy Current	Parameter Identification
**Decoupling/disentanglement**	3	1	1	3	3
**Multitask learning**	6	**0**	**0**	1	3
**Explainable/rule-based**	4	1	2	2	5
**Small-data/few-shot**	1	**0**	**0**	**0**	2
**Physics-informed**	1	**0**	**0**	**0**	2

**Table 6:** Yield strength and ductility of the tested steel classes, based on [67].

Steel Class	*Fyk* (MPa)	Ductility	*Euk* (%)	Rib Pattern
**A-I**	≥240	Highest	>5	Plain (smooth)
**A-III**	≥410	Lower	>5	Ribbed
**A-IIIN**	≥500	Lowest	>2.5	Ribbed, double-skew

**Table 7:** Factorial design of reinforced-concrete specimens: four combinations of rebar diameter (*D*) and steel class, each cast at five cover-thickness levels.

Steel Class/*D*	10 mm	12 mm
**A-I**	P1	X
**A-III**	X	P3
**A-IIIN**	P2	P4

**Table 8:** Median ± standard deviation of the signal amplitude-to-noise ratio (dimensionless—p.d.u.) for five cover-thickness levels, based on 20 repeated scans.

	20 mm	30 mm	40 mm	50 mm	60 mm
***B*** **_x_**	217 ± 50	132 ± 44	122 ± 38	152 ± 55	115 ± 85
***B*** **_y_**	104 ± 74	70 ± 33	74 ± 49	63 ± 42	44 ± 31
***B*** **_z_**	806 ± 221	468 ± 241	353 ± 209	267 ± 103	201 ± 71

**Table 9:** Worst-case standard deviations (SD_max_) and corresponding sensitivity thresholds sens.min for sensor signal discretization *k* = 4.

	Largest SD (p.d.u)	sens.min (p.d.u)
*B*_x_	85	340
*B*_y_	74	296
*B*_z_	241	963

**Table 10:** Association rules between signal features and structural parameters.

	*A*(*B_x_*)	*C*(*B_x_*)	*O*(*B_x_*)	*A*(*B_y_*)	*C*(*B_y_*)	*O*(*B_y_*)	*A*(*B_z_*)	*C*(*B_z_*)	*O*(*B_z_*)
***h*** ***↑***	↓100	↓100	**(-)100**	↓100	↓100	↓100	↓100	↓100	↑58
***D*** ***↑***	↑100	↓69	**(-)86**	↑100	↓83	↑100	↓91	↓65	↑100
***class*** ***↑***	↑94	(-)79	**↑95**	↓54	(-)58	↓57	↑99	(-)61	↑100

**Table 11:** Bin assignment for steel-class offset O(*B*_x_), before and after measurement correction.

Class	*n*	RAW	Corrected
***A-I***	100	97	100
***A-III***	100	96	100
***A-IIIN***	200	197	200

**Table 12:** OVL between adjacent 5 mm cover-thickness levels.

Pair (mm)	*OVL*	Pair (mm)	*OVL*
20–25	0.000	45–50	0.001
25–30	0.000	50–55	0.011
30–35	0.000	55–60	0.019
35–40	0.000	60–65	0.066
40–45	0.000	65–70	0.304

**Table 13:** In-sample and out-of-sample Root Mean Square Error (RMSE), and maximum absolute in-sample error, of estimated cover thickness from least-squares triangulation over the three simultaneous layer readings (15). Out-of-sample values are from leave-one-configuration-out cross-validation of Equation (14).

*h* (mm)	In-Sample RMSE (mm)	Out-of-Sample RMSE (mm)	Max. abs. Error, in-Sample (mm)
20	0.16	0.24	0.59
30	0.19	0.30	0.55
40	0.36	0.61	1.01
50	0.54	0.97	1.56
60	1.04	2.02	3.32

**Table 14:** OVL between Δ*x*(*D*_10_) and Δ*x*(*D*_12_), and Mann–Whitney U test results, by processing stage.

Processing	OVL	*P*
RAW	0.773	1.1 × 10^−3^
Median-filtered	0.671	2.1 × 10^−6^
GA-fitted	0.557	8.2 × 10^−7^
GA-fitted, averaged	0.531	6.8 × 10^−9^
Butterworth-filtered	0.614	7.1 × 10^−9^
Butterworth, averaged	0.544	1.3 × 10^−8^

**Table 15:** Rough-set approximation quality for diameter classification, by processing stage.

Processing	Bin Width	*α*_B_(*D*_10_)	*α*_B_(*D*_12_)	*γ*_B_(*P*)
RAW	1	0.033	0.083	0.058
Median-filtered	1	0.067	0.067	0.067
GA-fitted	0.2	0.467	0.300	0.383
GA-fitted, averaged	0.1	0.567	0.633	0.600
Butterworth-filtered	1	0.000	0.133	0.067
Butterworth, averaged	0.5	0.083	0.167	0.125

## Data Availability

The raw MFL measurement data supporting the findings of this study are available from the corresponding author upon reasonable request. The data are not publicly available due to administrative and institutional constraints regarding dataset ownership following an affiliation change.

## Supplementary Materials

The following supporting information can be downloaded at: https://www.mdpi.com/article/10.3390/s26217049/s1. **Figure S1.** PRISMA 2020 flow of identification, semantic ranking, shortlisting, and criteria-based narrowing. Blue boxes show identification and screening; grey boxes show intersections of criteria within the eligible set; the red box shows the number of research groups in the full intersection. **Figure S2.** Distribution of cosine distance between each record and the research-question embedding. The top-30 cut-off is marked. **Figure S3.** Enrichment of mean shared references and shared author keywords per record pair, as a function of cut-off depth. **Figure S4.** Concept composition of the top-30 shortlist compared with the full corpus. Shares are percentages of records within each set. **Figure S5.** Distribution of the analyzed records by: (a) country; and (b) journal. **Figure S6.** Annual output of the corpus. The 2026 bar is hatched because the year is partial (1 January–10 August 2026). **Figure S7.** Research landscape: (a) concept frequency in the corpus; (b) citation counts vs. cosine distance to the research question. **Figure S8.** Author-keyword co-occurrence network for terms appearing in at least three records, with Louvain clustering and node area proportional to record count. **Figure S9.** Distribution of authors by number of records, compared with the inverse-square Lotka prediction. **Figure S10.** Representation of approaches to each challenge in the full corpus (grey) and the reinforced-concrete subset (blue). Counts are records per approach; bold indicates challenges targeted in this study. The best-represented approach appears in at most four reinforced-concrete records. **Figure S11.** Average annual Google Trends search interest for the search terms “Small-data machine learning” and “Few-shot learning” (worldwide, 2004–2026). The 2026 value is partial (1 January–10 August 2026 only) [Google Trends]. **Figure S12.** Yearly publications on innovative NDT techniques for concrete, based on the Scopus database. The 2026 value is partial (1 January–10 August 2026 only) [SciVal]. **Figure S13.** Yearly publications on machine-learning techniques for predictive modeling, based on the Scopus database. The 2026 value is partial (1 January–10 August 2026 only) [SciVal]. **Figure S14.** Annual output of the 235-record corpus (grey) and of the 14 records addressing data scarcity (blue), with cumulative data-scarcity records on the right axis. The 2026 bar is hatched. **Table S1.** Bibliometric validation of the EmbedSLR shortlist. **Table S2.** Share of records (%) per period by method family. The 2026 portion of the last period is partial (to 10 August). Percentages are computed within each period, and do not sum to 100 because a record may carry several concepts. **Table S3.** Studies identifying reinforced-concrete or steel parameters by electromagnetic and magnetic NDT combined with computational inference.

## Abbreviations

The following abbreviations are used in this manuscript:

ACO	Amplitude–Correlation–Offset
AI	Artificial Intelligence
ARA	Association Rule Analysis
CNN	Convolutional Neural Network
CRISP-DM	Cross-Industry Standard Process for Data Mining
EMI	Electromagnetic Induction
GA	Genetic Algorithm
GPR	Ground-Penetrating Radar
IIR	Infinite Impulse Response
LSTM	Long Short-Term Memory
MFL	Magnetic Flux Leakage
ML	Machine Learning
MTL	Multitask Learning
NDE	Non-Destructive Evaluation
NDT	Non-Destructive Testing
OVL	Overlap Coefficient
PCA	Principal Component Analysis
RC	Reinforced Concrete
RMSE	Root Mean Square Error
RMSD	Root-Mean-Square Deviation
RQ	Research Question
SD	Standard Deviation
SHM	Structural Health Monitoring
SNR	Signal-to-Noise Ratio
SPM	Same-Pole Magnetization
SVM	Support Vector Machine
SVR	Support Vector Regression
TRL	Technology Readiness Level
XAI	Explainable Artificial Intelligence
YOLO	You Only Look Once
