# Supplementary material for: Feature-Level Decoupling of Reinforced-Concrete Parameters from Sparse MFL Data via Three-Layer Sensing
Source: Sensors (Basel). 2026 Nov 7;26(21):7049. doi: 10.3390/s26217049 (PMC13766382; doi:10.3390/s26217049)
Supplement: Supplementary file 1 [file sensors-26-07049-s001.zip › sensors-4594645-supplementary.pdf]

Supplementary Materials: *Feature-Level Decoupling of Reinforced Concrete Parameters from Sparse MFL Data via Three-Layer Sensing*

Frankowski, P.K.; Lasco, C.A.; Gatdula, A.K.S.; Mąka, M.; Ziętek, R.D.; Gordon, R.; Owczarek, P.; Pocheć, P.; Stawicki, T.; Matysik, S.

This document contains the full protocol and the complete bibliometric analysis of the systematic literature review that is summarized in Section 2.2 of the main text. Figure and table numbering is independent of the main manuscript, and is prefixed with S. Reference numbers follow the main manuscript.

S1. Systematic Literature Review Protocol

The systematic literature review (SLR) mapped the research landscape and identified the specific gap this work addresses. Scopus was selected for its broad journal coverage while excluding low-impact, poorly peer-reviewed literature; this choice was supported by EmbedSLR [1], the screening tool used in this review. The Scopus search was conducted on 13 August 2026, using a structured query, as follows:

The query is reproduced verbatim below, exactly as copied from the Scopus search-history interface:

```
( TITLE-ABS-KEY ( ( decoupl* OR disentangl* OR "multitask" OR "multi-task" OR "parameter
identification" OR "inverse problem" ) AND ( "machine learning" OR "deep learning" OR
"neural network" OR "artificial intelligence" ) AND ( NDT OR NDE OR nondestructive OR
"non-destructive" OR SHM OR "structural health" ) AND ( concrete OR rebar OR steel OR
magnetic OR "eddy current" OR MFL OR ultrasonic OR "ground penetrating radar" ) ) AND
PUBYEAR > 2004 )
```

The search, limited to publications after 2004, yielded 235 records: 165 articles, 53 conference papers, seven conference reviews, five reviews, three book chapters, one letter, and one editorial. No duplicates were found. Records were ranked semantically, using EmbedSLR [1], which embeds metadata and ranks by cosine distance to a research-question embedding, enabling efficient, reproducible screening without AI components.

For each record, the title, abstract, and keywords were embedded and ranked by cosine distance to the following natural-language statement of the research question:

> *"A machine-learning method that identifies multiple physical parameters of reinforced concrete (reinforcing-bar diameter, concrete cover thickness, and steel grade) from one-dimensional magnetic or electromagnetic non-destructive signals, by decoupling signal features so that each attribute (amplitude, offset, peak shift) responds to only one parameter, using interpretable rule-based models and requiring only a small number of reference measurements."*

The cosine distances ranged from 0.153 to 0.393; the 30 nearest records (≤ 0.244, well below the 0.3 threshold typically used to indicate semantic relevance) were retained for full screening.

Records were included if they met three criteria: a) applied a computational inference method; b) addressed NDT/NDE or SHM; and c) either operated on 1D measured signals or identified a physical parameter, rather than merely detecting a defect. Of the 30 shortlisted records, 22 met all criteria. The screening process followed the PRISMA 2020 guidelines (Figure S1).

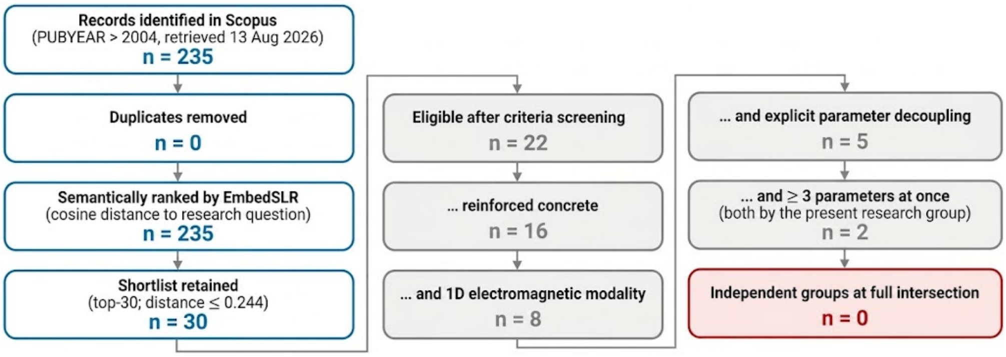

Figure S1. PRISMA 2020 flow of identification, semantic ranking, shortlisting, and criteria-based narrowing. Blue boxes show identification and screening; gra boxes show intersections of criteria within the eligible set; the red box shows the number of research groups in the full intersection.

S2. Validation of the Semantic Shortlist

The semantic shortlist was validated using two structural indices: mean shared references and author keywords, following the EmbedSLR protocol [1]. These embedding-independent metrics assess whether semantic proximity yields a coherent literature subset (Table S1).

Table S1. Bibliometric validation of the EmbedSLR shortlist.

Indicator	Top-30	Full corpus (n = 235)	Random 30 (2000 draws)	Enrichment vs. corpus
Shared references per pair	0.0667	0.0051	0.0056	13.0×
Jaccard index, references	0.00061	0.00009	0.00009	6.6×
Shared author keywords per pair	0.1655	0.0850	0.0867	1.9×
Jaccard index, author keywords	0.0145	0.0089	0.0091	1.6×

Table S1 shows that the top-30 shortlist has 13.0 times as many shared references per pair as the full corpus and 11.8 times as many as random 30-record draws ($p = 0.014$, 2000 draws). Shared author keywords are 1.9 times enriched. Jaccard indices show the same pattern (6.6× and 1.6× enrichment, respectively). These results confirm that the semantic ranking successfully identified a topically coherent subset.

Enrichment increases with stricter cut-offs: 121-fold at top-10, 29.8-fold at top-20, 13.0-fold at top-30, dropping to 4.8-fold at top-50 and 1.05-fold at top-150 (Figure S3). This decrease indicates

enrichment results from ranking, not a corpus artifact, and that topical coherence declines notably, beyond 50 records.

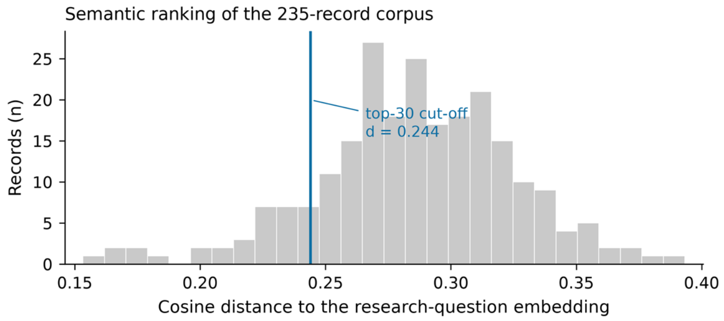

Figure S2. Distribution of cosine distance between each record and the research-question embedding. The top-30 cut-off is marked.

Figure S2 shows the cosine distances between each record's embedding and the research-question embedding, measuring semantic dissimilarity from 0 to 1. Lower values mean closer topical alignment; values under ~0.3 suggest topical convergence. The top-30 records for analysis, marked on the plot, are below 0.25, confirming they are the most semantically aligned studies.

Figure S3 shows how the two structural indices vary with cut-off depth. Semantic proximity indicates topical alignment, shared references reveal scholarly links, and author keywords show how authors frame their work. For the top-30 shortlist, shared references are enriched 13.0-fold and author keywords 1.9-fold, relative to the full corpus, significantly above random draws ($p = 0.014$). Enrichment declines monotonically with depth, confirming that the top-30 cut-off balances topical focus against sample size.

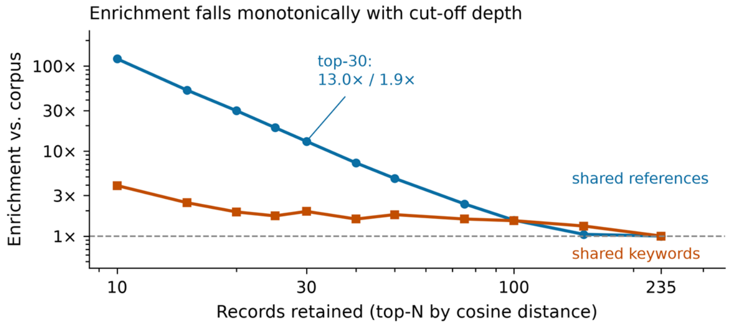

Figure S3. Enrichment of mean shared references and shared author keywords per record pair, as a function of cut-off depth.

The shortlist's concept composition (Figure S4) indicates that the semantic ranking effectively selected studies aligned with the research domain. The shortlist is enriched for reinforced concrete, multitask learning, and XAI, while NDT-specific methods and simulation-focused records are less prevalent, compared with the full corpus.

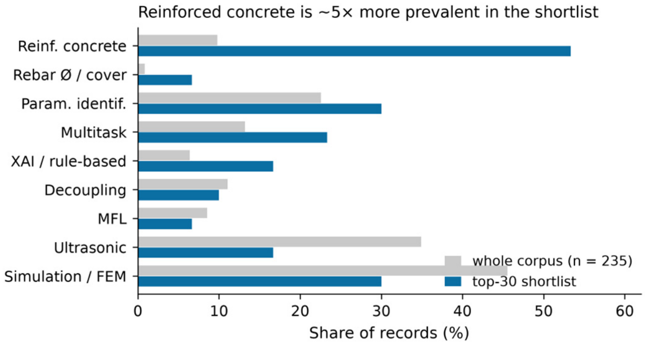

Figure S4. Concept composition of the top-30 shortlist compared with the full corpus. Shares are percentages of records within each set.

Figure S5 presents the geographical distribution of the analyzed records by country (a) and distribution by journal (b). China and the United States together account for nearly 60% of the corpus, indicating a concentration of research activity in these countries.

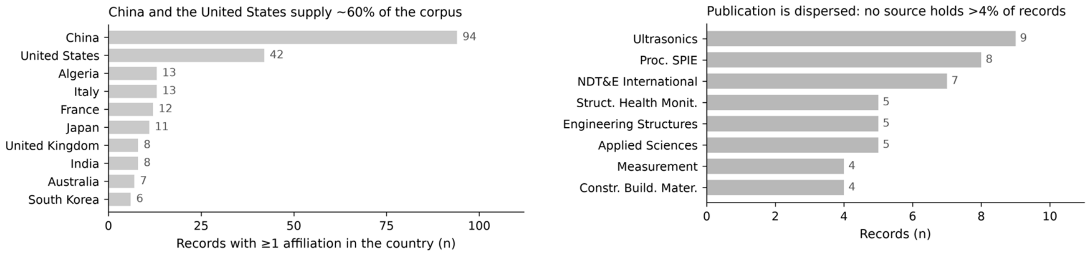

Figure S5. Distribution of the analyzed records by (a) country; and (b) journal.

S3. Structure and Dynamics of the Field

Analysis of the annual output confirms the conclusions from Figure 1 and Figure 2. Annual output increased from 3.4 records per year during 2005–2009 to 24.0 in 2021–2025, a 7.1-fold increase. As of 10 August 2026, 47 records have already been indexed for 2026 (Figure S6). Table S2 shows growing interest in multitask learning, decoupling, and XAI/rule-based methods, with a focus on computational approaches.

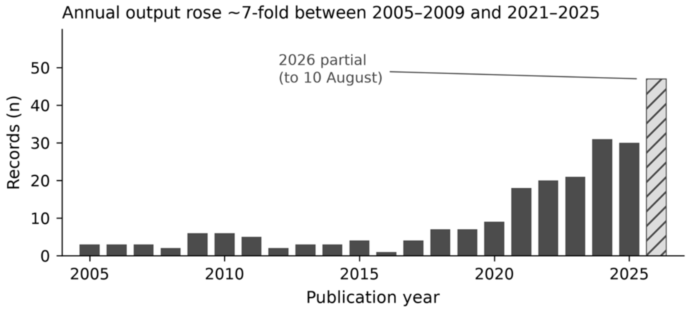

Figure S6. Annual output of the corpus. The 2026 bar is hatched because the year is partial (1 January – 10 August 2026).

Among computational approaches, the concepts behind this study have emerged recently: no records involving multitask learning before 2015, and, by 2025–2026, their share reached 24.7%, explicit decoupling 20.8%, and explainable or rule-based approaches 15.6%. This shows significant potential for further growth.

Table S2. Share of records (%) per period by method family. The 2026 portion of the last period is partial (to 10 August). Percentages are computed within each period, and do not sum to 100 because a record may carry several concepts.

Period	N	Multitask learning	Decoupling	XAI / rule-based
2005–2009	17	0.0	0.0	0.0
2010–2014	19	0.0	5.3	0.0
2015–2019	23	4.3	4.3	0.0
2020–2022	47	2.1	2.1	2.1
2023–2024	52	19.2	13.5	3.8
2025–2026	77	24.7	20.8	15.6

S4. Research Landscape and the Quantified Gap

Figure S7 shows two views of the research landscape. Panel (a) displays the absolute frequency of concepts in the corpus. Key concepts (multitask learning, decoupling, XAI/rule-based methods, small-data learning, and rebar) are among the least frequent, despite recent growth (Table S2). Panel (b) plots citation counts against cosine distance to the research-question embedding. The most semantically aligned records are recent and mostly uncited, except for a few near the research question, which receive above-average citations, indicating a small, but valuable, niche. These insights highlight the scarcity and under-representation of research on interpretable, data-efficient decoupling in RC inspection.

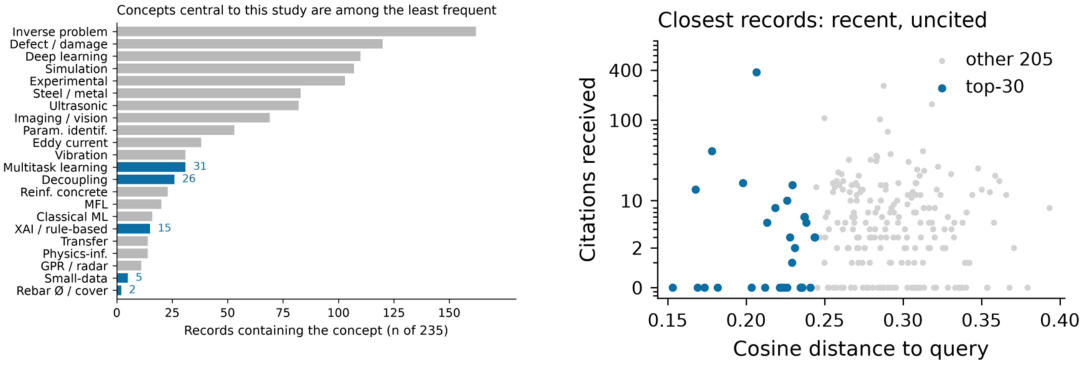

Figure S7. Research landscape: (a) concept frequency in the corpus; (b) citation counts vs. cosine distance to the research question.

The author-keyword co-occurrence network (Figure S8) shows a comparable separation between method families: no cluster links parameter decoupling to reinforcement geometry.

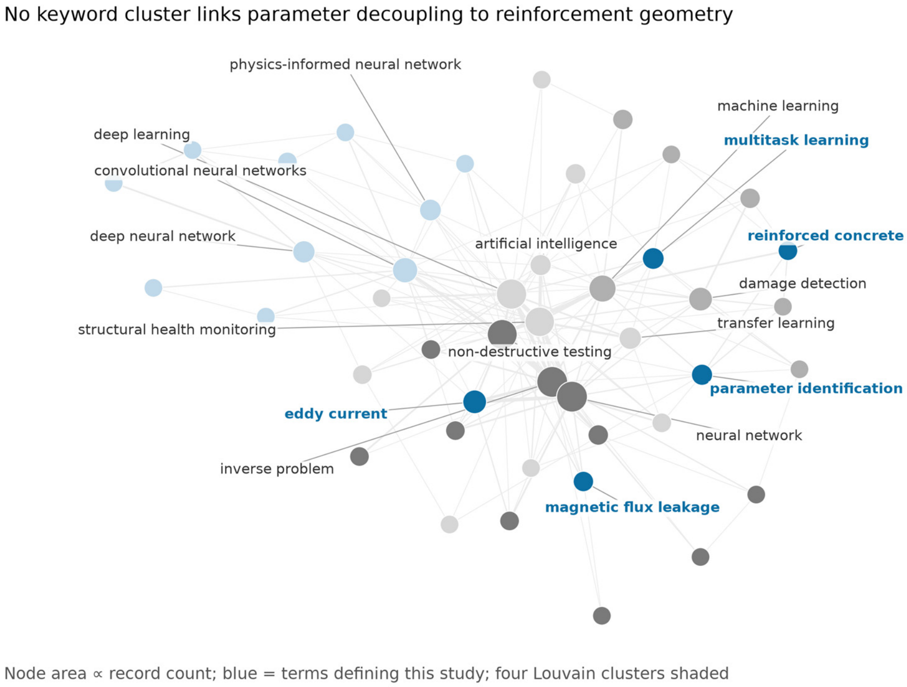

Figure S8. Author-keyword co-occurrence network for terms appearing in at least three records, with Louvain clustering and node area proportional to record count.

Authorship is highly dispersed: 88% of authors in the corpus contribute a single record, well above the share predicted by the inverse-square Lotka law (Figure S9). Together with the co-occurrence matrix reported as Table 6 of the main text, this indicates an unexplored niche, rather than a saturated research area.

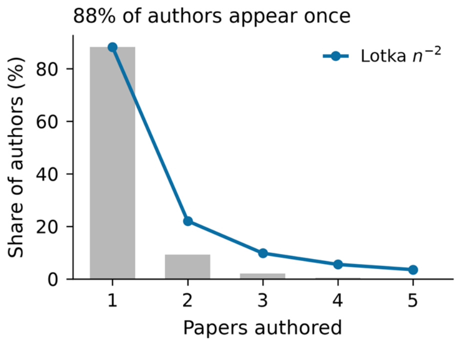

Figure S9. Distribution of authors by number of records, compared with the inverse-square Lotka prediction.

S5. Coverage of the Six Challenges in the Corpus

Figure S10 quantifies how much of the reviewed corpus supplies a remedy for each of the six challenges listed in Table 3 of the main text, and how little of that remedy reaches reinforced-concrete studies, specifically.

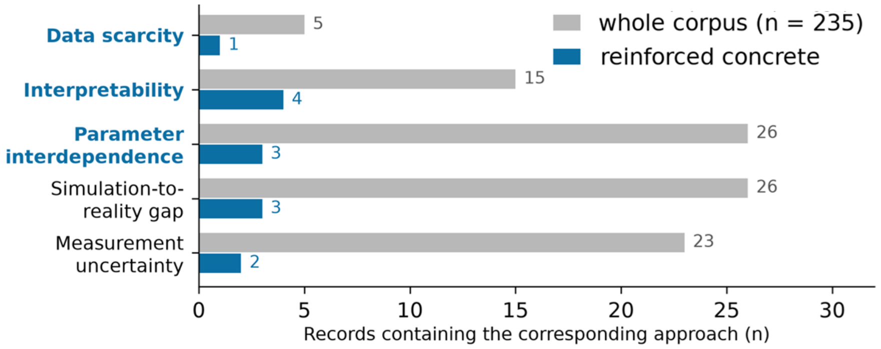

Figure S10. Representation of approaches to each challenge in the full corpus (gray) and the reinforced-concrete subset (blue). Counts are records per approach; bold indicates challenges targeted in this study. The best-represented approach appears in at most four reinforced-concrete records. Section S6: Research-interest and publication trends (Figures S11–S13); Section S7: Study-by-study survey of parameter identification from electromagnetic and magnetic NDT (Table S3); Section S8: Technology-readiness constraints (Figures S11–S13).

S6. Research-Interest and Publication Trends

The figures below document the two premises stated in a single sentence in Section 1.1 of the main text: that interest in small-data and few-shot learning has grown steadily since 2004, and that publication output on innovative NDT methods for concrete has more than tripled over the same period, increasingly involving artificial intelligence. They are reported here, rather than in the main text, so that the evidence remains verifiable without occupying the Introduction.

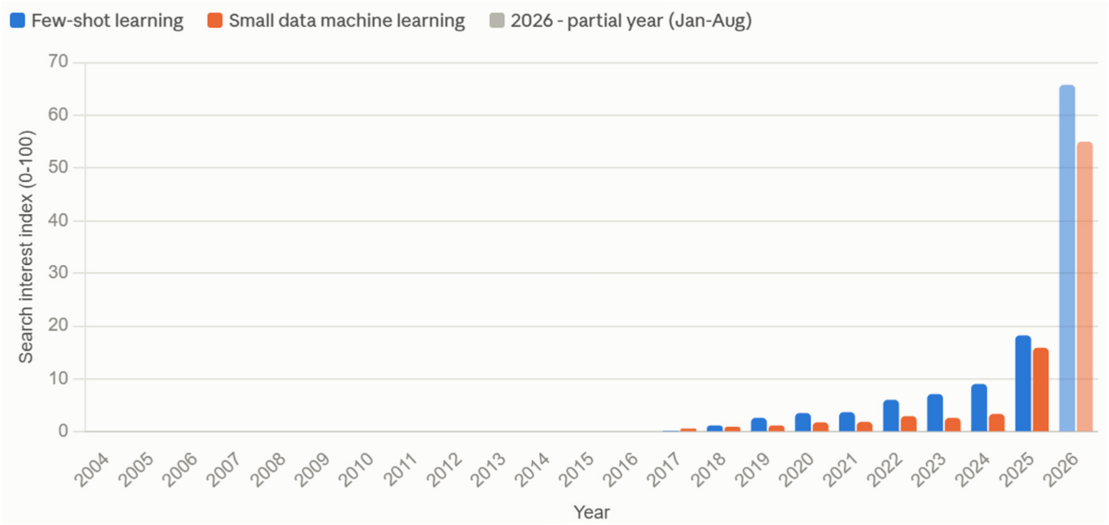

Figure S11. Average annual Google Trends search interest for the search terms “Small-data machine learning” and “Few-shot learning” (worldwide, 2004–2026). The 2026 value is partial (1 January–10 August 2026 only) [Google Trends].

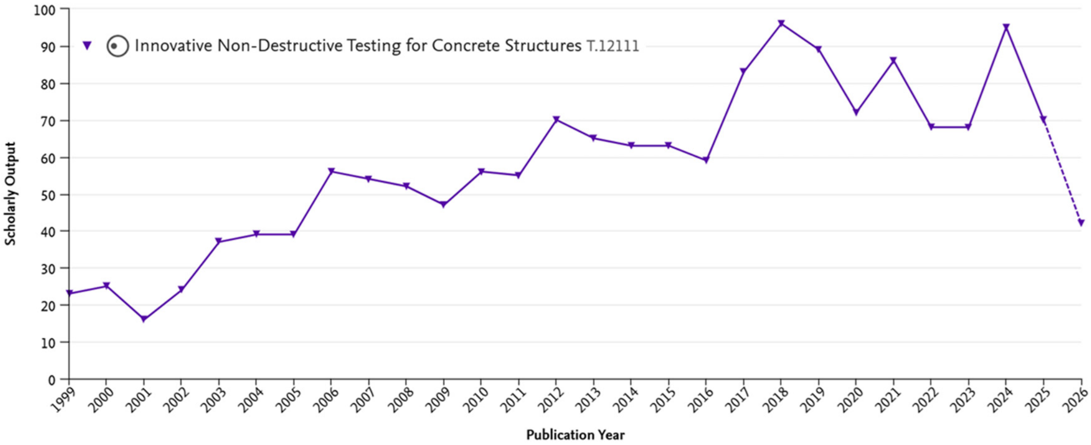

Figure S12. Yearly publications on innovative NDT techniques for concrete, based on the Scopus database. The 2026 value is partial (1 January–10 August 2026 only) [SciVal].

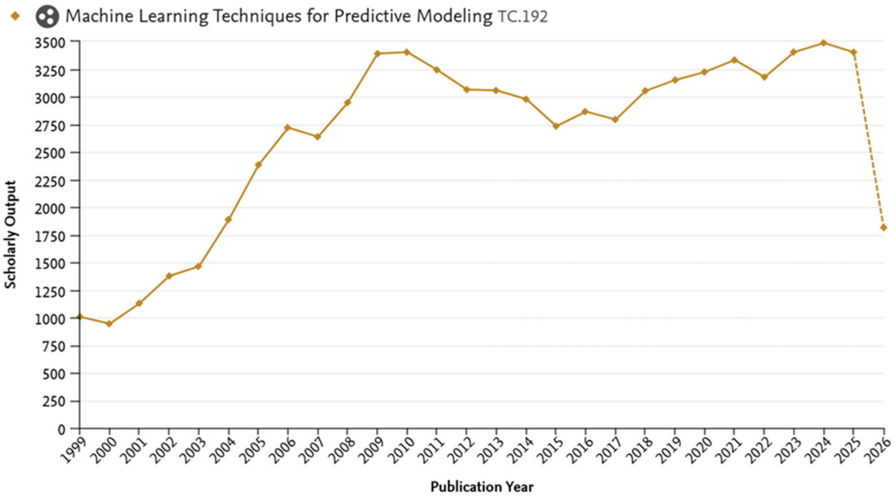

Figure S13. Yearly publications on machine-learning techniques for predictive modeling, based on the Scopus database. The 2026 value is partial (1 January–10 August 2026 only) [SciVal].

These two observations do not conflict with the scarcity of small-data methods reported in Figure 2 of the main text. Figure S11 measures worldwide search interest across all research fields, whereas the main-text count measures how many records within the NDT corpus actually apply such methods. A method may be widely adopted in general, and still be almost absent from one application domain; that mismatch is the gap this study addresses. Figure S14 quantifies this mismatch within the corpus itself: only 14 of the 235 records address data scarcity, and their share rose only modestly, from 1.7% in 2005–2019 to 7.4% in 2020–2026, even as annual output grew almost ninefold. Worldwide search interest (Figure S11) and in-corpus practice (Figure S14) therefore diverge sharply.

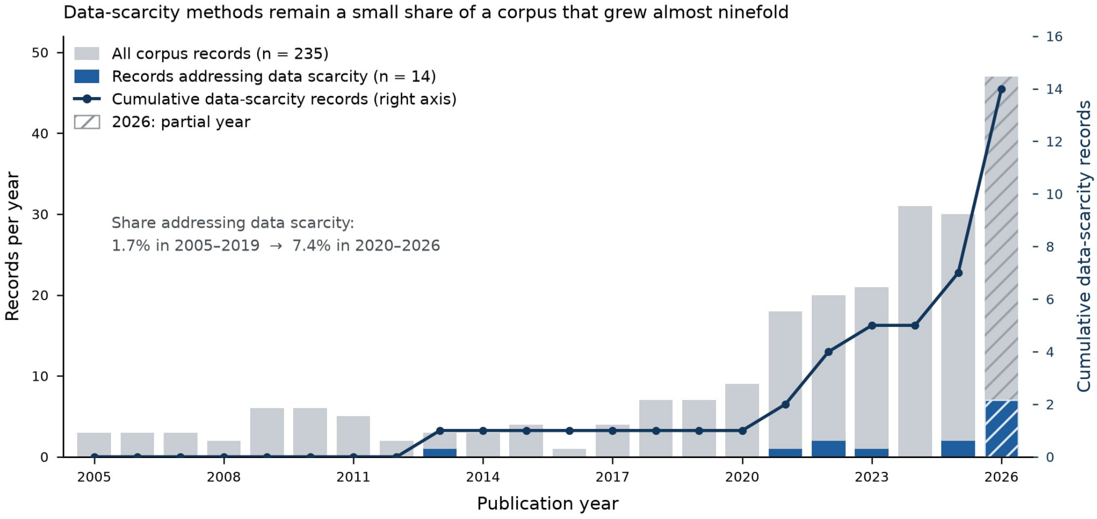

Figure S14. Annual output of the 235-record corpus (gray) and of the 14 records addressing data scarcity (blue), with cumulative data-scarcity records on the right axis. The 2026 bar is hatched because the year is

partial (1 January–10 August 2026). The share of data-scarcity records rose from 1.7% in 2005–2019 to 7.4% in 2020–2026.

S7. Parameter Identification from Electromagnetic and Magnetic NDT: Study-by-Study Survey

The table below surveys studies that identify reinforced-concrete or steel parameters from electromagnetic and magnetic NDT signals combined with computational inference. It is reported here, rather than in the main text, where the two gaps it documents are stated in a single sentence in Section 1.2.3: no study recovers three coupled parameters simultaneously, and none combines parameter decoupling with transparent, human-readable rules.

Table S3. Studies identifying reinforced-concrete or steel parameters by electromagnetic and magnetic NDT combined with computational inference.

Study	Modality	Method	Parameters recovered	Performance	Ref
Sbartaï 2009	GPR	ANN	Moisture, chloride	Two parameters; validated on laboratory slabs	[47]
Travassos 2009	GPR	Parametric and non-parametric inversion	Inclusion geometry, position	Two parameters	[48]
Cacciola 2009	Electromagnetic	Neuro-fuzzy	Fissure reconstruction	One parameter	[49]
Queiroz 2013	GPR	Feature relevance	Reinforcement vs. fissure	One parameter	[26]
Zhou 2018	GPR + EMI	Model-based	Rebar diameter, concrete cover thickness	Two parameters; cover error < 5%	[46]
Giannakis 2021	GPR	NN + random forest	Rebar diameter	One parameter; synthetic training data	[32]
Li 2022	GPR + EMI	YOLO v3 + 1D-CNN	Rebar diameter, concrete cover thickness	Two parameters; autonomous estimation	[27]
Maciusowicz 2022	Barkhausen noise	Time–frequency + ML	Steel class	One parameter; classification	[50]
Huang 2023	Eddy current	ML decoupling	Permeability, conductivity, thickness, lift-off	Four parameters; in model space	[40]
Khedr 2025	GPR	YOLO v8	Rebar diameter, steel class	97.2% accuracy in a building case study	[28]
Zhang 2026	Pulsed eddy current	BC-TSN two-stage net	Rebar diameter, concrete cover thickness	Two parameters; RMSE 0.91 mm (diameter), 0.87 mm (cover)	[34]
This work	MFL, eddy current	ACO + association rules	Rebar diameter, concrete cover thickness, steel class	Three parameters; feature-level decoupling; few reference measurements	[17] [23]

S8. Technology-Readiness Constraints

Section 3.8 of the main text states the technology-readiness level reached by this study. The constraints behind that level, and the path beyond it, are set out below.

Preparing reinforced-concrete specimens with controlled parameter variations and repeated multi-layer MFL scans is time-intensive, which inherently limits the number of combinations a single study can investigate. The focus is therefore not on a deployable field instrument, but on demonstrating two scientific contributions: (I) the value of multi-layer sensors with varying lift-off for parameter decoupling, and (II) the need for explainable AI in NDT. Rather than treating AI as an opaque black box, the proposed approach frames it as an interpretable, trustworthy glass box.

Like many NDT studies, this work is constrained by the time and resources required for specimen preparation and measurement; covering the full parameter space with classical methods would demand a multi-year effort. The present study therefore prioritizes simple, interpretable methods that reduce this effort. By establishing transparent, rule-based relationships between signal features and physical parameters, the approach offers a foundation for future field-deployable systems that can be refined as more data become available. This aligns with the broader vision of explainable AI in engineering, where transparent models are essential for validation and stakeholder confidence [10][11]. The glass-box principle is particularly relevant in safety-critical domains such as NDT infrastructure monitoring [13], and explainability supports adoption by enabling engineers to verify results [77]. The Limitations Section discusses detailed limitations and steps toward higher TRL levels.
